# Applications and insights from continuous dengue virus infection in a stable cell line

**DOI:** 10.3389/fimmu.2025.1618650

**Published:** 2025-06-24

**Authors:** M. Jane Morwitzer, Ying Yi Zheng, Heather Friberg, Jeffrey R. Currier

**Affiliations:** Department of Virus-Host Interactions, Viral Diseases Program, Walter Reed Army Institute of Research, Silver Spring, MD, United States

**Keywords:** dengue, DENV, serotype, DC-SIGN, CEM.NK^R^, immunoassay, dengue vaccines, dengue therapeutics

## Abstract

Dengue is caused by the four serotypes of dengue virus (DENV-1-4) and poses a significant global public health challenge, with an estimated 100–400 million infections annually. Severe dengue manifestations, such as Dengue Hemorrhagic Fever (DHF) and Dengue Shock Syndrome (DSS), are influenced by immune responses, particularly during secondary infections with different serotypes. Antibody-dependent enhancement (ADE) of DENV infection is a critical mechanism in dengue immunopathogenesis, underscoring the need for comprehensive evaluation of antibody responses. Traditional cell lines used for DENV propagation exhibit variability and present logistical challenges for assessing non-neutralizing antibody functions. Here, we report the establishment of a stable CEM-NK^R^ cell line expressing DC-SIGN, designated CEM2001, capable of supporting continuous infection with all four DENV serotypes. These cell lines allow for continuous DENV infection, enabling detailed immunoassays to evaluate serotype-specific and cross-reactive non-neutralizing antibody responses. Our approach offers a significant advancement in dengue research, providing a consistent and reliable system to study DENV immune responses and supporting future efforts to develop and evaluate dengue therapeutics and vaccines.

## Introduction

Dengue, a mosquito-borne disease caused by four dengue virus serotypes (DENV-1-4), has seen a dramatic increase in incidence in recent decades, currently putting about half of the world’s population at risk, with an estimated 100–400 million infections occurring annually (1–3). Due to its prevalence, which extends to at least 128 countries and is predicted to expand geographically, dengue poses a major global public health problem (4, 5). While many dengue cases are asymptomatic or self-limiting, up to 500,000 individuals develop severe dengue disease each year, classified as either Dengue Hemorrhagic Fever (DHF) or Dengue Shock Syndrome (DSS). DHF/DSS is characterized by rapid capillary leak, thrombocytopenia, and altered hemostasis, with the host immune response being a crucial factor. DHF/DSS patients exhibit elevated proinflammatory cytokine and chemokine levels, referred to as a cytokine storm, leading to vascular integrity breakdown, particularly during secondary infections with a different DENV serotype in older children and adults (3, 6–11).

A major risk factor for developing severe disease is the presence of pre-existing immunity to the primary DENV serotype that is recalled during a secondary infection with a heterologous serotype. Current evidence indicates antibody-dependent enhancement (ADE) of DENV infection as a central mechanism in the immunopathogenesis of dengue (1, 12). Cross-serotype-reactive antibodies generated during a primary infection can wane over time and become sub-neutralizing at lower levels, binding but not neutralizing DENV. Ultimately ADE of infection can occur, wherein virus-antibody complexes facilitate Fc receptor-mediated enhanced entry into target cells, resulting in enhanced replication and increased cellular viral load (13). An increasing spatial and temporal overlap in the circulation of multiple DENV serotypes in many endemic areas underscores the need for safe and efficacious tetravalent countermeasures for dengue (2, 14). Notably, the only FDA-approved vaccine to date, Dengvaxia^®^, has restricted indication due to a lack of efficacy and a potential for increased risk of severe dengue in seronegative individuals (15). More recently, Qdenga^®^ (TAK-003) has been approved in several endemic and non-endemic countries for use regardless of prior dengue infection status, expanding vaccine access to broader populations (16).

Antibodies play a crucial role in host defense against pathogens through a variety of mechanisms (17). Beyond neutralization, Fc-mediated effector functions such as antibody-dependent cellular cytotoxicity (ADCC), antibody-dependent complement deposition (ADCD), and antibody-dependent cellular phagocytosis (ADCP), involve the Fc region interacting with effector cells or molecules to induce cytotoxic effects on infected cells, thereby driving antiviral control (18–25). Since ADE during heterologous dengue infection can occur (10), assessment of the quality of antibodies—including both neutralizing and non-neutralizing effector functions—elicited by DENV infection or vaccination across all four serotypes is critical for understanding protective versus enhancing antibody responses against dengue. This is especially important given the lack of a clearly defined neutralizing antibody correlate of protection, and emerging evidence suggesting that Fc-mediated effector functions may also contribute to protective immunity (25–29).

Cell lines such as Vero and C6/36 have been used extensively for viral propagation and *in vitro* viral assays (25, 30–40), however infecting cells with a certain multiplicity of infection (MOI) for each iteration of an assay has several pitfalls that can affect the consistency and reliability of experimental results. One key issue is variability in infection efficiency. Factors such as the exact MOI, handling of the virus stock, and condition of the cells can all introduce variability. It is also logistically challenging to ensure that the cells are at an optimal state for infection and that virus stocks are consistently prepared, adding layers of complexity regarding timing and coordination. In addition, having to infect cells for each assay substantially increases the workload and complexity of each experiment and can be particularly burdensome for high-throughput studies or long-term experiments. Furthermore, the continuous need for fresh virus stocks can require frequent virus stock preparation and quickly deplete resources, making the approach costly and time-consuming. In contrast, a stable cell line that can sustain continuous DENV infection would avoid many of the above issues and provide a more consistent and reliable system for studying anti-DENV antibody function. The use of authentically infected cells, rather than cells transfected with individual viral proteins, enables the assessment of anti-viral functions in the context of *de novo* transcription, translation and protein trafficking.

Despite the significant potential of continuous infection cell lines for studying DENV and antibody responses, current research typically relies on repeated individual infections or stable cell lines expressing one viral antigen such as nonstructural protein 1 (NS1) (41–45). Persistent DENV-2 infection has previously been documented in K562, Raji, and HSB-2 cell lines (46), however, a stable cell line capable of continuous infection across all four DENV serotypes for use in immunoassays to characterize antibodies has not yet been reported. Here, we successfully established a stable CEM-NK^R^ cell line expressing dendritic cell-specific intercellular adhesion molecule 3-grabbing non-integrin (DC-SIGN), designated CEM2001, capable of supporting continuous infection with all four serotypes of dengue virus (DENV1-4). This cell line provides a robust platform for facilitating comprehensive and high-throughput investigations into serotype-specific and cross-reactive anti-DENV antibody responses. Utilizing these continuous cultures, we can perform detailed immunoassays to assess the binding and effector functions of DENV-specific antibodies. This approach represents a significant advancement in dengue research, offering a valuable tool for studying DENV immune responses through effector mechanisms such as opsonization, phagocytosis, and complement activation, and provides a scalable platform for evaluating antibody functions elicited by infection or vaccination.

## Materials and methods

### Human plasma and peripheral blood mononuclear cell isolation

DENV-naïve human blood was obtained through a healthy blood donor protocol maintained at the Walter Reed Army Institute of Research (WRAIR protocol #2567.03). Peripheral blood mononuclear cells (PBMCs) and plasma were separated from heparinized whole blood by a standard Ficoll-Paque procedure. Separated plasma was stored at -80°C and isolated PBMC were cryopreserved and stored in the vapor phase of liquid nitrogen. WRAIR #2567.03 was reviewed by the WRAIR Human Subjects Protection Branch and granted a Non-Human Subjects Research determination. Informed consent was obtained from all participants. DENV-immune plasma was sourced commercially from the AccuSet™ Dengue Performance Panel (SeraCare, Milford, Massachusetts). Prior to use all plasma samples were heat-inactivated at 56°C for 30 minutes and clarified by centrifugation at 13,000 × *g* for 10 minutes. **Table 1** lists all critical reagents used in this study together with supplier names and catalog numbers.

**Table 1:** List of all critical reagents used in this study.

Item	Source/Manufacturer	Catalog/Material Number
Accuset™ Dengue Performance Panel	SeraCare Life Sciences Inc.	0845-0143 (Batch #10375776)
Geneticin sulfate (G - 418)	Life Technologies (Gibco)	10131-027
Penicillin/Streptomycin	ThermoScientific	15140122
L - glutamine	ThermoScientific	25030081
Fetal bovine serum	Corning	35-016-CV
RPMI 1640	ThermoScientific (Corning)	10-040-CM
Phosphate buffered saline - PBS 1X pH 7.4	Gibco	10010-023
BD Phosflow Permeabiliztion Wash Buffer I	BD Biosciences	557885
BD GolgiStop	BD Biosciences	51-2092KZ
BD GolgiPlug	BD Biosciences	51-2301KZ
MINI26-1KT	Sigma-Aldrich	MKCQ0938
PKH26GL-1KT	Sigma-Aldrich	MKCQ6477
Aqua Live/Dead stain	ThermoScientific	L34597-A
Low-Tox Guinea Pig complement	Cedarlane	CL4051
Gelatin Veronal Buffer	Sigma-Aldrich	G6514
20% Formaldehyde	Tousimis	1008B
Mouse anti-CD209 (human) AF647 (Clone: 9A9E8)	Biolegend	330112
Mouse anti-CD209 (human) purified (Clone: 9A9E8)	Biolegend	330102
Rat anti-CD209 (human) purified (Clone: A20120B)	Biolegend	380402
Mouse anti-CD209 (human) purified (Clone: DCS-8C1)	Biolegend	343002
Mouse anti-CD4 (human) purified (Clone: RPA-T4)	Biolegend	300502
Mouse anti-CD317 (human) purified (Clone: RS38E)	Biolegend	348402
Mouse IgG1𝒌 (Clone: MOPC-21)	Biolegend	400166
Mouse IgG2A𝒌 (Clone: MG2a-53)	Biolegend	401508
Rat IgG2B𝒌 (Clone: R35-38)	BD Biosciences	555845
Goat anti-Human IgG AF647 (polyclonal)	Southern Biotech	2040-31
Goat anti-Mouse Ig BV421 (polyclonal)	BD Biosciences	563846
Goat anti-Rat Ig BV421 (polyclonal)	BD Biosciences	565013
anti-CD14 (human) AF700 (Clone: M5E2)	BD Biosciences	557923
Goat anti-C3 (guinea pig) FITC (polyclonal)	MP Biomedicals	55385

### Monoclonal antibodies

Hybridoma cell lines for monoclonal antibodies (MAbs) 2H2, 4G2, 7E11 and 3H5 are maintained at WRAIR. Commercially available monoclonal antibodies for DC-SIGN (CD209), CD4, CD14, CD317 and all isotype control MAbs, were sourced as listed in **Table 1**. The variable regions of the previously described VDB MAb collection (32) were codon-optimized, synthesized *in vitro*, and subcloned into an expression vector containing the human IgG1-Fc region by a commercial source (Genscript Inc. Piscataway, NJ). The expression vector was then transfected into Chinese Hamster Ovary (CHO) cells, and the resulting IgG was purified from the cell culture supernatant using the MabSelect SuRe™ LX affinity matrix. Antibody purity was verified by sodium dodecyl sulfate-polyacrylamide gel electrophoresis (SDS-PAGE), and the final concentration was determined by measuring absorption at 280 nm.

### Cell line establishment and maintenance

All cell lines were maintained in RPMI 1640 medium (Corning, 10-040-CV) supplemented with 10% fetal bovine serum (FBS) (Corning 35-016-CV), 1% penicillin/streptomycin, and 1% L-glutamine (referred to as R10) under standard culture conditions at 37°C in 5% CO_2_.

**Figure 1:**
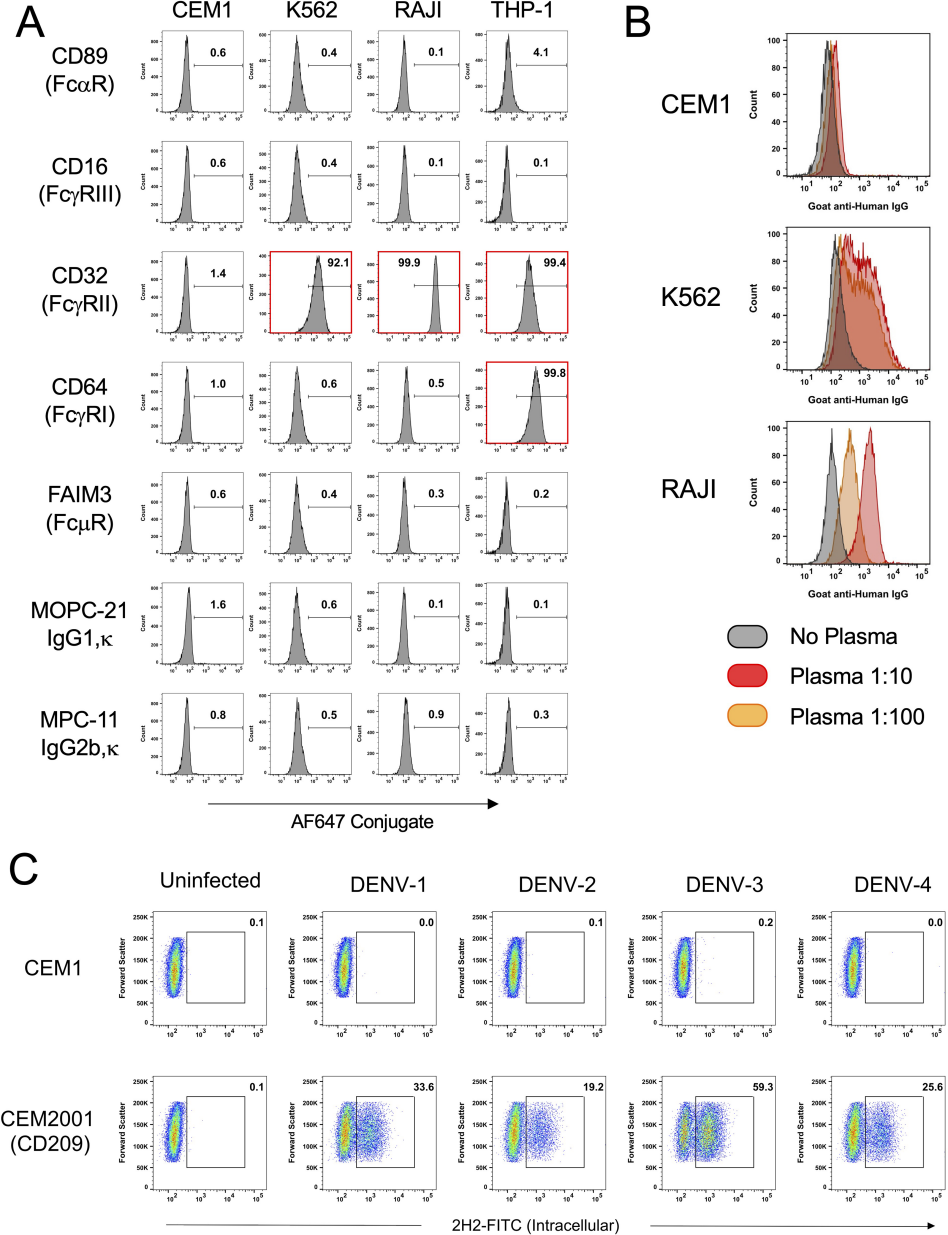
The CEM1 cell line displays a paucity of surface expressed FcRs, a corresponding low level of IgG binding from human plasma, and when transfected to express human DC-SIGN (CD109), is readily infectable with all four serotypes of DENV. **(A)** CEM1, K562, RAJI and THP-1 cells were assessed for surface expression using monoclonal antibodies specific for FcαR (CD89), FcγRI (CD64), FcγRII (CD32), FcγRIII (CD16) and FcμR (FAIM3). K562, RAJI and THP-1 cells express high levels of CD32, while THP-1 cells also express CD64. In contrast, CEM1 cells express none of the common FcRs on their surface. **(B)** As predicted by the relative expression levels of CD32, RAJI (DMFI at 1:10 = 1851) and K562 (DMFI at 1:10 = 909) cells display substantial levels of IgG binding when incubated with healthy human donor plasma, whereas CEM1 (DMFI at 1:10 = 71) cells show a correspondingly low level of IgG binding from healthy human donor plasma. **(C)** The CEM2001 cell line (DC-SIGN transfected CEM1 cells; see **Supplementary Figure 1**) is permissive to infection by all four serotypes of DENV. Percent infection was assessed 24 hours after incubation with 5 PFU per cell of each serotype.

The parental CEM.NK^R^ cell line is a T-lymphoblastoid cell line that lacks appreciable surface expression of canonical Fc receptors, including FcγRI (CD64), FcγRII (CD32), and FcγRIII (CD16), as confirmed by flow cytometry (**Figure 1A**). This FcR^NULL^ phenotype minimizes background antibody binding and makes the line ideal for assessing Fc-mediated effector functions without confounding Fc-FcR interactions. In addition, CEM.NK^R^ cells are resistant to direct lysis by natural killer (NK) cells and do not stimulate monocyte activation, further supporting their use as an unbiased platform for antibody functional assays.

A DC-SIGN (CD209)-expressing cell line was generated by electroporating a sub-clone of the CEM.NK^R^ cell line with a linearized pcDNA3.1(+) plasmid containing the codon-optimized sequence of DC-SIGN (GenBank NM_021155.4), followed by G-418 (Geneticin™ Sulfate) selection. Stable clones were generated by sorting for CD209^HIGH^-expressing cells using a BD FACSAria Fusion instrument into 96-well plates. A clone was selected for expansion based on stable and uniform CD209 expression (See **Supplementary Figure 1**). This clone was designated CEM2001.

### Viruses

The following DENV strains were used in this study and are maintained in the Viral Diseases Program at WRAIR: DENV-1 (strain WestPac74; GenBank U88535), DENV-2 (strain S16681; GenBank GU289914), DENV-3 (strain CH53489; GenBank DQ863638), and DENV-4 (strain TVP-360; GenBank KU513442). Stocks of each were derived from C6/36 or VERO cell propagation.

### Continuous infection culture

CEM2001 cells were infected at an MOI = 0.01 with each DENV serotype to initiate infection. 5 × 10^6^ cells were infected in 1 mL of R10 in a 15 mL snap-top polypropylene tube. Cells were incubated in a shaker incubator for 2 hours at 37°C, 8% CO_2_, 135 rpm. After incubation, cells were washed three times in R10 and centrifuged at 400 × *g* for 5 minutes. Cells were then placed in a T75 flask with a volume of 25 mL R10 and incubated at 37°C 5% C0_2_ for 3 days. To maintain continuous cultures for 45 days without replenishment of uninfected cells, cells were split 8:1 and 16:1 every 3–4 days. Infection was monitored using intracellular flow staining with 2H2-FITC MAb from day 14 to day 45. Replenishment cultures were initiated by a split of day 18 continuous cultures using uninfected CEM2001 added back to infected CEM2001 at a 4:1 ratio of uninfected to infected cells. Replenishment was performed on days 16, 25, 35, and 42 using 1 × 10^6^ infected cells replenished with 4 ×10^6^ uninfected cells (5 × 10^6^ total). In between replenishments, cells were split 4:1 and 8:1 every 2–3 days as required. Replenishment cultures were cryopreserved at day 42 to establish stocks of continuous passage 1 and subsequently at passages 3, 5 and 8.

### DC-SIGN blocking assay

To quantify inhibition of cell-cell spread of virus, uninfected CEM2001 cells were incubated with three different anti-DC-SIGN antibodies before incubation with continuously infected cells. Uninfected cells were seeded at 50,000 cells per well in a 96-well plate and pre-incubated with MAbs for 30 minutes at 37°C in 100 μL R10. Infected cells were then added at 2,500 cells per well (100 μL R10) to a total volume of 200 μL R10 per well. Plates were incubated at 37°C 5% CO_2_ for 72 hours and then stained for intracellular 2H2 expression with FITC-labeled MAb. Flow cytometry was performed with 20,000 cells per well acquired based on light scattering gating and percent viral inhibition was determined. Half-maximum virus inhibition (IC_50_) was determined by applying an asymmetric, five parameter, non-linear regression curve fit using GraphPad Prism software (version 8.4.3).

### Surface (opsonization) and intracellular antibody staining

Cell lines were stained for surface antigen expression and intracellular antigen expression as follows. All surface staining application incubations were performed at 4°C, while intracellular staining was performed at 20°C (ambient room temperature) in 96-well plates. All centrifugation steps (cell washing) were performed at 800 × *g* for 3 minutes and all staining volumes were 100 μL per well with 1 × 10^5^ cells per well. For surface staining cells were dispensed in 96 well round-bottomed plates, washed twice with Flow Wash Buffer (FWB; PBS plus 2% FBS) and incubated for 30 minutes with primary MAb or plasma diluted in FWB to the indicated concentrations. Secondary fluorophore conjugated antibodies, either goat anti-mouse or goat anti-human diluted in FWB, were then added to the cells and incubated for 20 minutes. Cells were washed twice and either fixed with 2% formaldehyde in FWB and kept at 4°C prior to data acquisition by flow cytometry or fixed with 4% formaldehyde in PBS for 30 minutes at room temperature prior to intracellular staining. For intracellular staining, fixed cells were pelleted and subsequently resuspended in Permeabilization Wash Buffer (PWB; BD Biosciences) containing MAb directly conjugated with fluorophore. Cells were incubated with PWB and MAb at room temperature for 30 minutes, then washed twice with FWB. Cells were then analyzed by flow cytometry using a BD LSR Fortessa SORP flow cytometer.

### Antibody-dependent phagocytosis assay

Cryopreserved PBMCs were thawed, seeded at 250,000 cells per well in 96-well round-bottom plates, and rested overnight at 37°C. The next day uninfected and DENV-1, -2, -3 and -4 infected CEM2001 target cells were stained with a 1:500 dilution of PKH26 in Diluent C (Millipore Sigma) according to the manufacturer’s instructions. Target cells were then incubated with sera at the indicated dilution or MAbs (10 μg/mL) for 15 minutes at 37°C in a 96-well format. After antibody incubation, target cells were centrifuged at 400 × *g* for 5 minutes at room temperature and the supernatant was discarded before being added to 250,000 PBMCs/well. The cells were incubated for 1 hour at 37°C and were subsequently washed twice, stained with anti-human CD14-AF700 and Live/Dead Aqua stain in PBS, and then analyzed on a BD LSR Fortessa SORP flow cytometer.

### Antibody-dependent complement deposition assay

Uninfected and DENV-1, -2, -3 and -4 infected CEM2001 target cells (1 × 10^5^ per well) were incubated with plasma (1:25 dilution in R10) in a 96-well round-bottom plate for 30 minutes at 37°C, 5% CO_2_ to allow antibody opsonization. Cells were then washed twice with PBS (1X, pH 7.4) and incubated with Low-Tox Guinea Pig complement (Cedarlane) diluted 1:50 in Gelatin Veronal Buffer (Millipore Sigma) for 20 minutes at 37°C, 5% CO_2_ to enable classical pathway-mediated complement deposition. Following incubation, excess complement was removed by centrifugation at 800 × *g* for 3 minutes at 4°C. Cells were washed once with PBS and stained with FITC-conjugated goat anti–Guinea Pig Complement C3 antibody (MP Biomedicals) diluted 1:100 in PBS for 20 minutes at room temperature. After two additional PBS washes, cells were fixed with 4% formaldehyde in FWB for 15 minutes. Fixed cells were washed once with PWB, incubated for 15 minutes at room temperature in PWB, and then stained for intracellular DENV antigen using 2H2 MAb conjugated to APC (diluted 1:500 in PWB) for 30 minutes. Cells were washed twice with FWB and analyzed by flow cytometry on a BD LSR Fortessa SORP flow cytometer. All wash steps were performed by centrifugation at 800 × *g* for 3 minutes at 4°C.

## Results

### Target cell line selection

This study aimed to develop a continuous DENV infection culture system and utilize it to evaluate viral inhibition and characterize DENV-specific antibodies. As shown in **Figure 1A**, in comparison with other common suspension cell lines used for DENV research the CEM1 clone retains the FcR^NULL^ phenotype of the parental CEM-NK^R^ cell line. The benefit of this phenotype is demonstrated clearly in **Figure 1B**. Normal human plasma (NHS) from a healthy donor was incubated at varying dilutions with CEM1, K562, or RAJI cells. CEM1 displayed no appreciable binding of IgG at dilutions as high as 1:10, whereas K562 and RAJI cells demonstrated human IgG binding down to a dilution of 1:100. This feature precludes the use of K562, RAJI and most likely any other FcR expressing cells as substrate cell lines for opsonization and other antibody effector function assays due to high “background” binding of IgG. Therefore we used CEM1 cells to establish a stable cell line expressing DC-SIGN (**Supplementary Figure 1**), a major attachment factor that facilitates DENV entry into host cells (47), and demonstrated its ability to be infected by all four DENV serotypes (**Figure 1C**). Incubation of this new cell line, CEM2001, with 5 PFU per cell of a representative isolate of each serotype resulted in robust infection with all four serotypes.

### Target cell line characterization

The CEM2001 DENV infection system was then subjected to a series of experiments to determine suitability as an authentic representation of an immunologically relevant virus-infected target cell. We first assessed whether antibody binding to infected cells was due to *de novo* expressed, viral proteins and glycoproteins on the surface of the infected cell. Cells were infected with 50 PFU per cell of each serotype of DENV for 2 hours followed by treatment with or without trypsin prior to incubation for a total of 24 hours. Trypsin-treatment was performed to remove any residual virions binding directly to DC-SIGN on the target cells.

**Figure 2:**
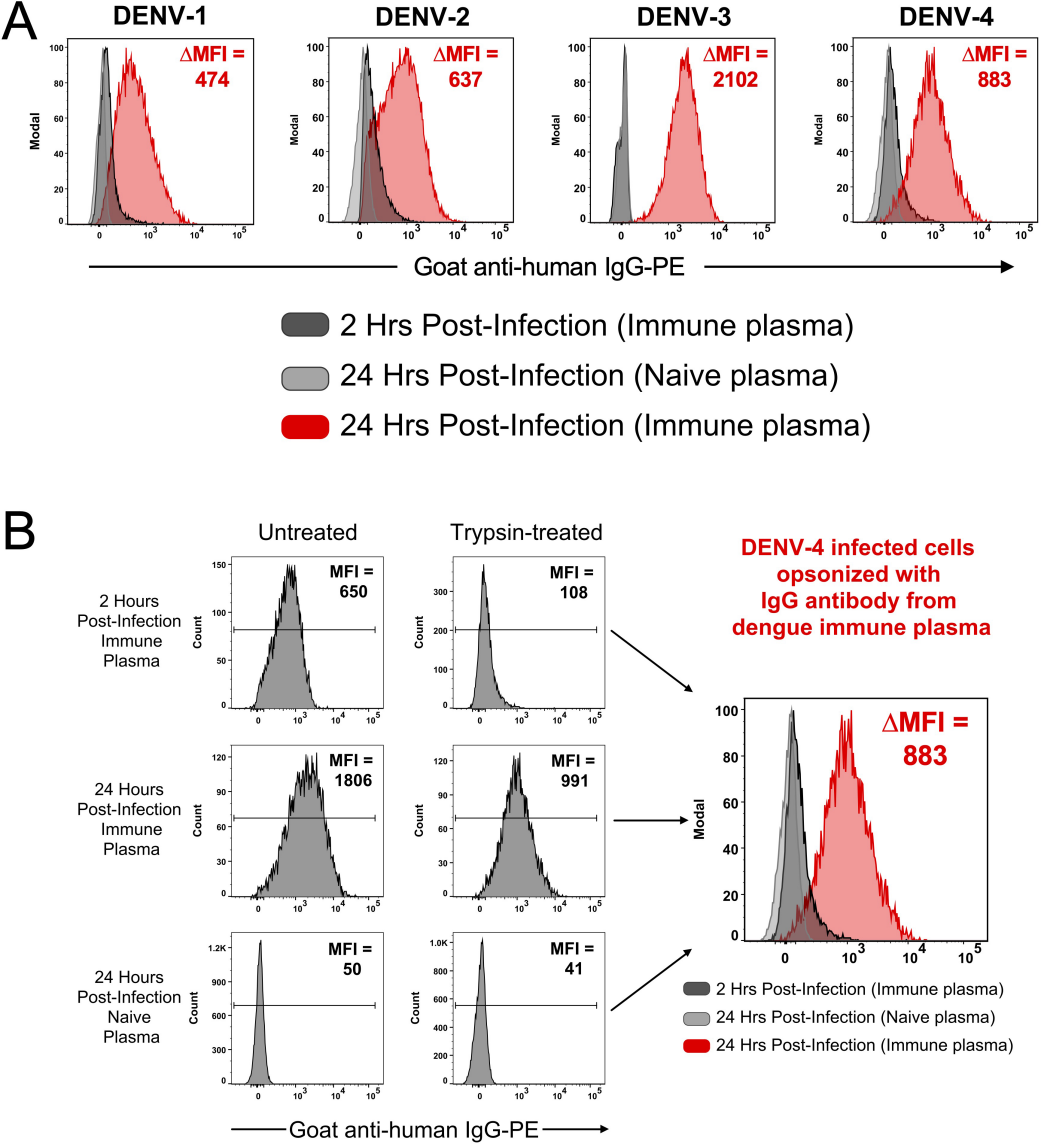
CEM2001 cells were infected for 2 hours with 50 PFU per cell of each of the serotypes of DENV and then treated, or not, with Trypsin/EDTA and incubated for a further 24 hours. **(A)** Surface antigen expression was assessed using DENV-immune plasma and a secondary goat anti-human IgG antibody. A clear increase in *de novo* surface antigen expression occurs for all four serotypes from 2 hours post-infection to 24 hours post-infection. No antibody-binding (opsonization) occurs when DENV-naïve plasma is used. **(B)** The gating strategy used for DENV-4 is depicted. Some binding of virions (from the inoculum) directly to the surface of the DC-SIGN positive cell line is evident at 2 hours, however trypsin treatment clearly removes these virions. The subsequent increase in antigen expression at 24 hours is quantified as the DMFI. **Supplementary Figure 2** shows the equivalent gating strategy and data for DENV-1, DENV-2 and DENV-3.

As shown in **Figure 2A** cells were stained at 2 hours and 24 hours post-infection for IgG binding (1:100 plasma dilution) by DENV-immune or DENV-naïve plasma. For all four serotypes, no appreciable antibody binding occurs with immune plasma at 2 hours, or with naïve plasma at 24 hours. A substantial shift in fluorescence intensity for IgG-binding occurred with immune plasma at 24 hours. **Figure 2B** shows all staining conditions for trypsin-treated DENV-4 infected cells shown in **Figure 2A**. For non-trypsin treated DENV-4 infected cells, IgG binding is detected at 2 hours post-infection with immune plasma, but this is substantially reduced with trypsin treatment (ΔMFI = 542). Clear evidence of post-infection expression of viral protein products on the surface on the infected cell is shown by the MFI increase between 2 and 24 hours (ΔMFI = 883) in the presence of immune plasma only. **Supplementary Figure 2** shows equivalent staining strategies and *de novo* expression of viral protein products on the surface on the infected cells for DENV-1 (ΔMFI = 474), DENV-2 (ΔMFI = 637) and DENV-3 (ΔMFI = 2102). Although these ΔMFI values varied across serotypes, this likely reflects differences in antigen trafficking, surface abundance, or epitope accessibility rather than a direct difference in infectivity. All infections in this experiment were performed using a fixed viral input (50 PFU per cell); however, intracellular infection levels were not normalized across serotypes at this timepoint. Therefore, these values should be interpreted as a qualitative confirmation of *de novo* surface antigen expression rather than a precise, quantitative comparison of antigen load between serotypes.

**Figure 3:**
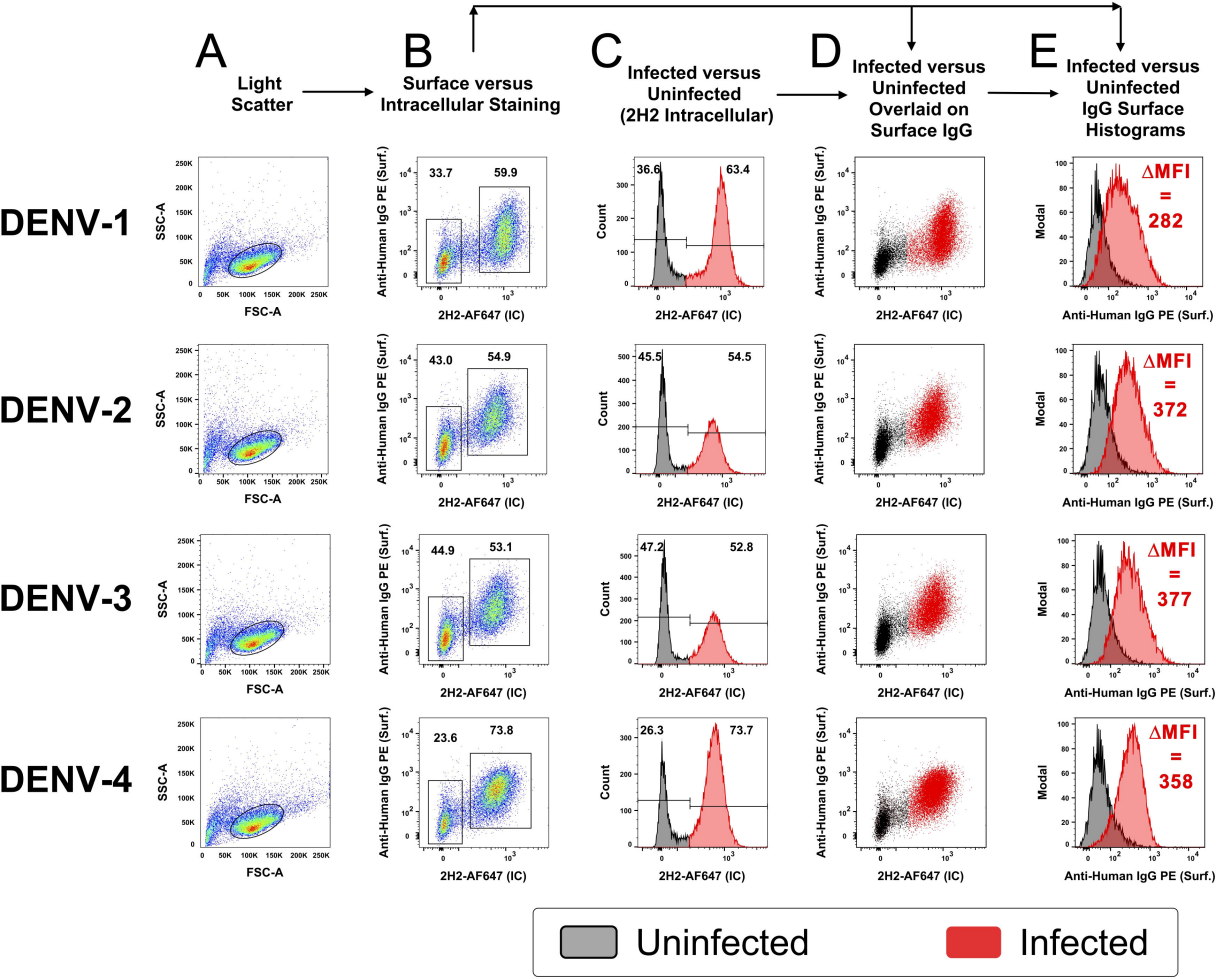
Opsonizing antibody binding is restricted to DENV-infected cells. CEM2001 were infected with serial dilutions of DENV to reach approximately 50% of cells infected, then stained for intracellular 2H2 expression (marker of infection), and surface IgG binding from DENV-immune plasma. Columns **(A–E)** progressively show the gating strategy for light scatter, identification of infected cells and an overlay of cell infection versus IgG opsonization with DENV-immune plasma. For each of the four serotypes of DENV, infected cells (2H2-positive) were shaded in red while uninfected are shaded black. There is a clear preferential binding of IgG from DENV-immune plasma to infected cells versus uninfected cells. The increase in IgG binding is quantified as the DMFI. **Supplementary Figure 3** shows a complete outline of the gating strategy for each serotype with uninfected cultures and DENV-naïve plasma included as controls.

To verify that IgG antibodies from immune plasma were binding to infected cells in the culture and not to virions that were exiting an infected cell and then binding to surrounding cells in a paracrine manner, infections were performed with lower infectious dosages. CEM2001 cells were infected with 5 PFU per cell and serially diluted to result in an MOI of ~0.5 (~50% of cells infected). After 24 hours cells were surface stained for IgG using DENV-immune plasma and stained intracellularly with AF647-labelled 2H2 MAb (anti-prM of all DENV-serotypes) (**Figure 3**). To control for variation in infection frequency, IgG binding was analyzed specifically within the 2H2^+^ (infected) population for each serotype. In this controlled analysis, the ΔMFI values across DENV-1 through DENV-4 were relatively similar (282, 372, 377, and 358, respectively), suggesting consistent levels of surface antigen expression per infected cell under matched input conditions.

**Figure 4:**
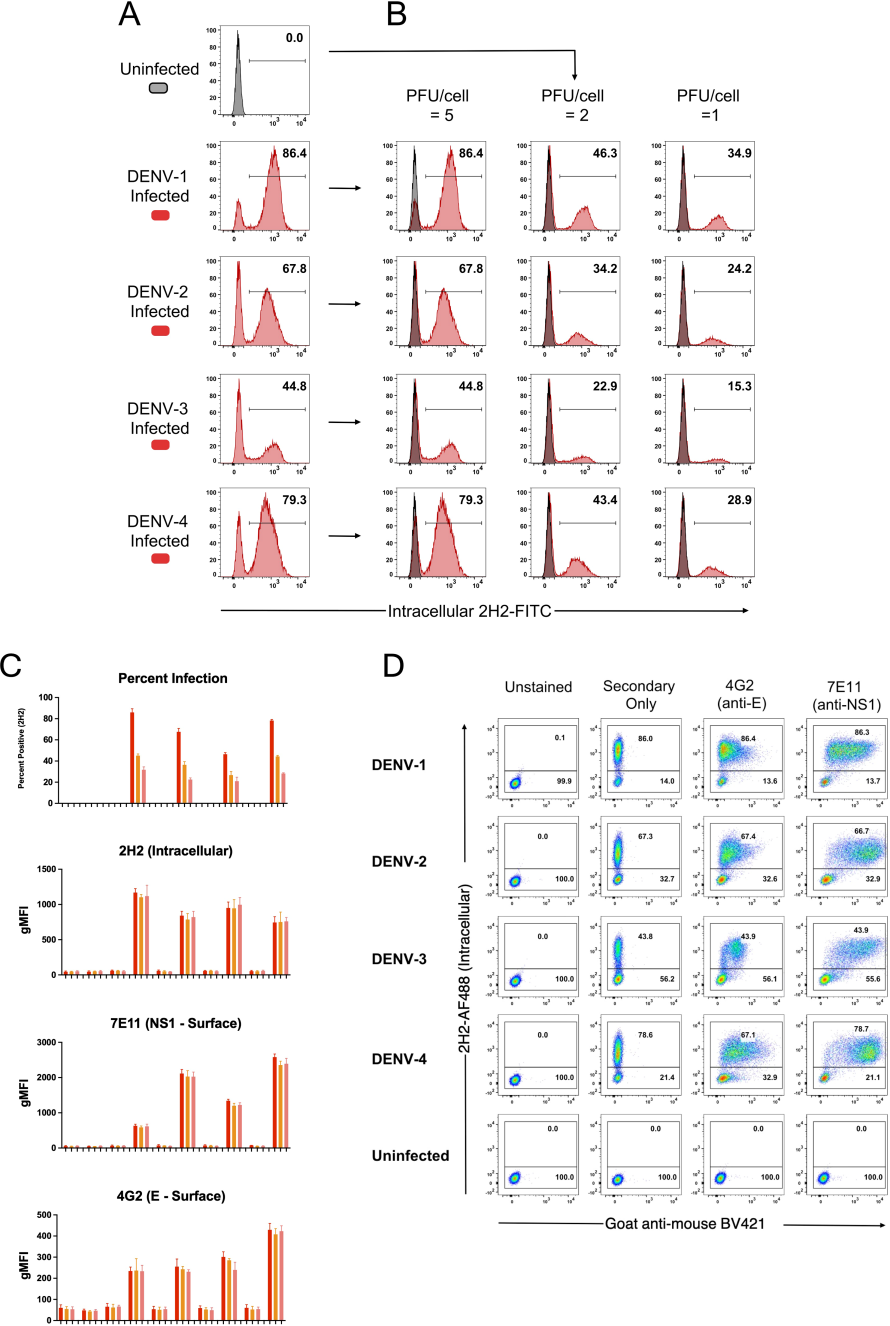
Viral antigen expression remains constant as the force of infection varies. CEM2001 cells were infected with titrated amounts (PFU per cell) of each DENV serotype and stained for intracellular 2H2 expression and surface NS1 (7E11 MAb) and surface E expression (4G2 MAb). **(A, B)** Intracellular 2H2 expression was assessed at 24 hours post-infection with each of the four serotypes of DENV (5 PFU per cell). Uninfected control cells were overlayed for each infection culture and presented as histograms. 2H2 negative cells clearly overlap with the control cell peak. **(C)** Surface expression of E and NS1 antigen was assessed for different levels of viral infection of target cells. As the force of infection deceases (percent infection) the MFI of intracellular 2H2 remains the same for each serotype, while surface expression of E (4G2) and NS1 (7E11) remains constant per cell. **(D)** The gating strategy for detecting surface expression of DENV E protein and NS1 glycoprotein is shown for all four serotypes. Control staining, secondary goat anti-mouse BV421 staining, as well as 4G2 and 7E11 are shown in dot-plots against intracellular 2H2 (directly conjugated 2H2-AF488).

Clearly, cells positive for prM-expression, and hence DENV infection, displayed discrete and high-level surface binding and opsonization with IgG compared to 2H2-negative (uninfected) cells. **Supplementary Figures 3A–D** shows comparative analyses for each serotype of naïve and immune plasma for infected and uninfected control cultures. **Supplementary Figure 4** demonstrates that intracellular expression of prM greatly exceeds its surface expression on infected cells and is a useful indicator of cell infection. Furthermore, as the infectious dose of virus on the target cell is decreased and the frequency of infected cells drops, the amount of viral antigen detected per infected cell (as determined by MFI) remains relatively stable (**Figures 4A–C**). Of note is that both NS1 (detected with MAb 7E11) and E protein (detected with MAb 4G2) derived from the infecting virus appears on the cell surface at high levels in a 24-hour culture (**Figure 4D**). Hence, a single infectious unit of virus results in similar amounts of viral antigen production per cell, both intracellularly and on the surface.

### Development of continuous DENV-1–4 infected cell cultures

**Figure 5:**
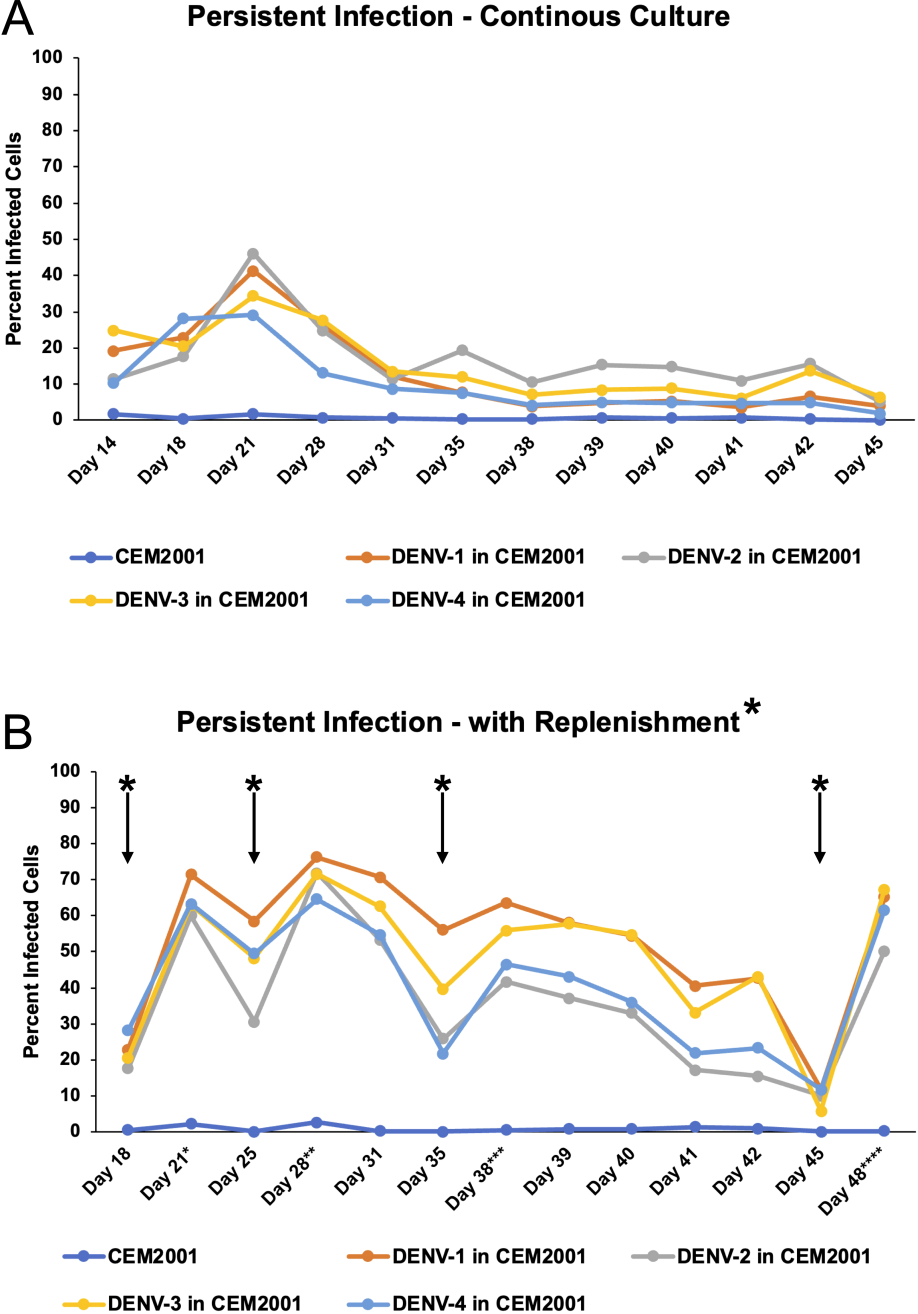
DENV can be maintained in CEM2001 cells in long-term cultures using an uninfected cell replenishment strategy. **(A)** CEM2001 cells were infected with all four serotypes of DENV and then maintained in continuous culture for 45 days. The level of infection was determined using intracellular 2H2 staining. **(B)** The continuous cultures above were split at day 18 and replenished with uninfected CEM2001 at days indicated by an asterisk* (days 18, 25, 35 and 45). An add back ratio of 4:1 uninfected cell to infection culture cell ratio was used.

**Figure 6:**
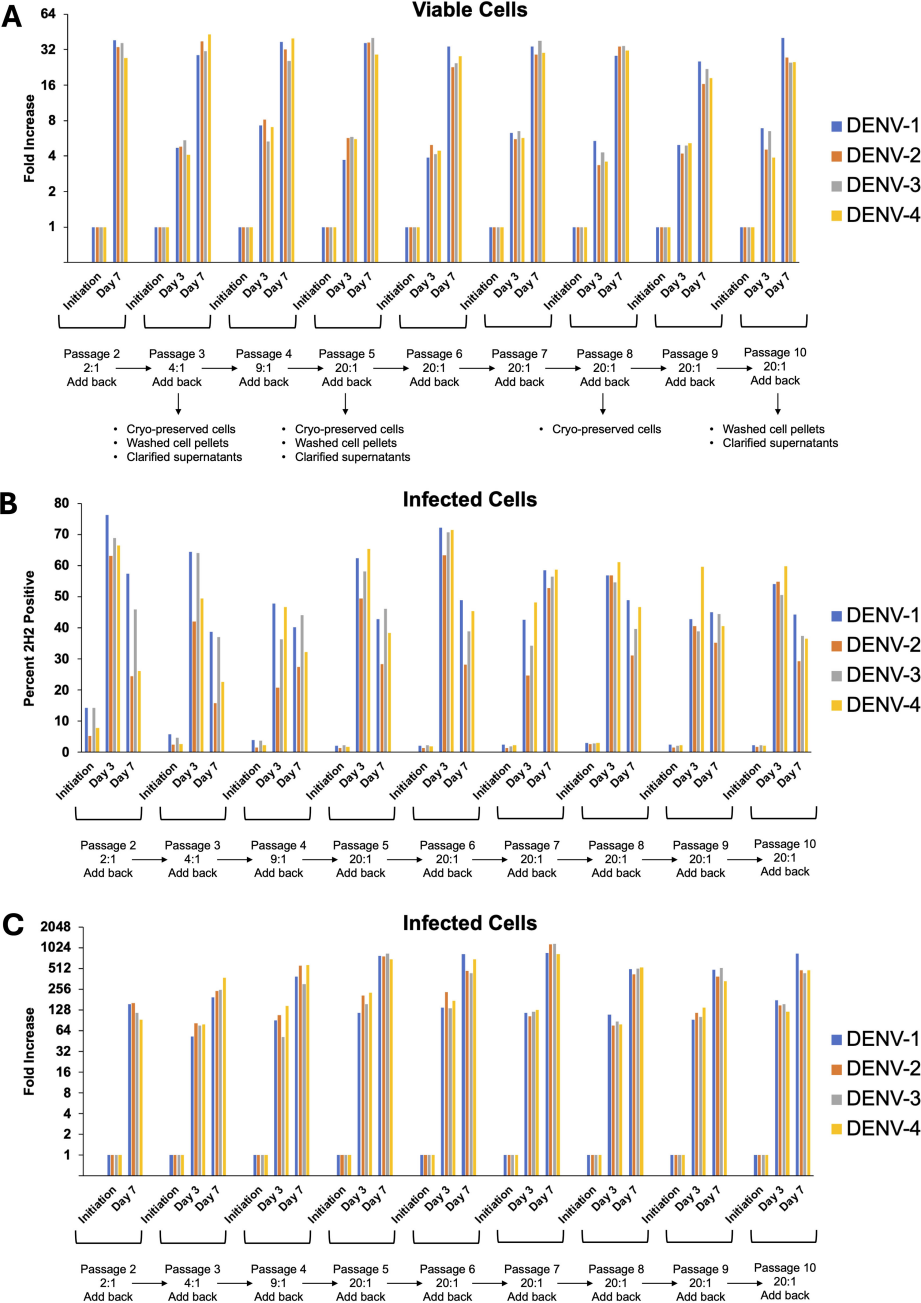
DENV can be maintained in CEM2001 cells through 10 passages of weekly replenishment and retains remarkably similar cell and virus replication kinetics. Infected cells from day 42 of the replenishment infection cultures (**Figure 5B**) were considered “passage 1” with virus adapted to growth in the CEM cell background. Nine further passages of weekly replenishment were performed for each serotype with the replenishment ratio denoted. The following parameters were assessed at initiation, day 3 and day 7 of each passage: **(A)** Fold increase in total viable cells for each serotype; **(B)** Percent infected cells for each serotype; **(C)** Fold increase in infected cell number for each serotype.

After confirming infection using a traditional transient, short-term infection-based method, we sought to establish a continuous culture of DENV-1–4 infected cells. Initiating infection at an MOI of 0.01, we observed continuous viral spread in each serotype’s culture that peaked around day 21 for all four serotypes (**Figure 5A**). Following this peak, the number of infected cells plateaued then gradually declined. To sustain higher DENV infection levels, we tested whether replenishment with uninfected cells could maintain the infection. Adding uninfected cells resulted in rapid infection and an increase of infected cells, which remained high for at least 7 days (**Figure 5B**). Successive replenishments showed that DENV cultures potentially could be maintained indefinitely. An add-back uninfected to infected cell ratio of 20:1 was determined to be the most practical ratio to use (**Supplementary Figure 5**). Using a 7-day cycle for infection and replenishment, cultures were maintained through 10 cycles (passages) and total cell count, infected cell count, and percentage of infected cells were monitored for all four serotypes at days 3 and 7 (**Figures 6A–C**). Cell growth kinetics, and virus infection and spread in the cultures were remarkably consistent for all four serotypes through passage 10. Of note is that an increase of total infected cell numbers of over 500-fold was consistently obtained in 7-day cultures for all four serotypes. The gating strategy for flow cytometric assessment of post-replenishment infected cells opsonized with dengue-immune versus dengue naïve plasma is presented in **Supplementary Figure 6**.

### Microwell adaptation for measuring direct inhibition of viral spread

**Figure 7:**
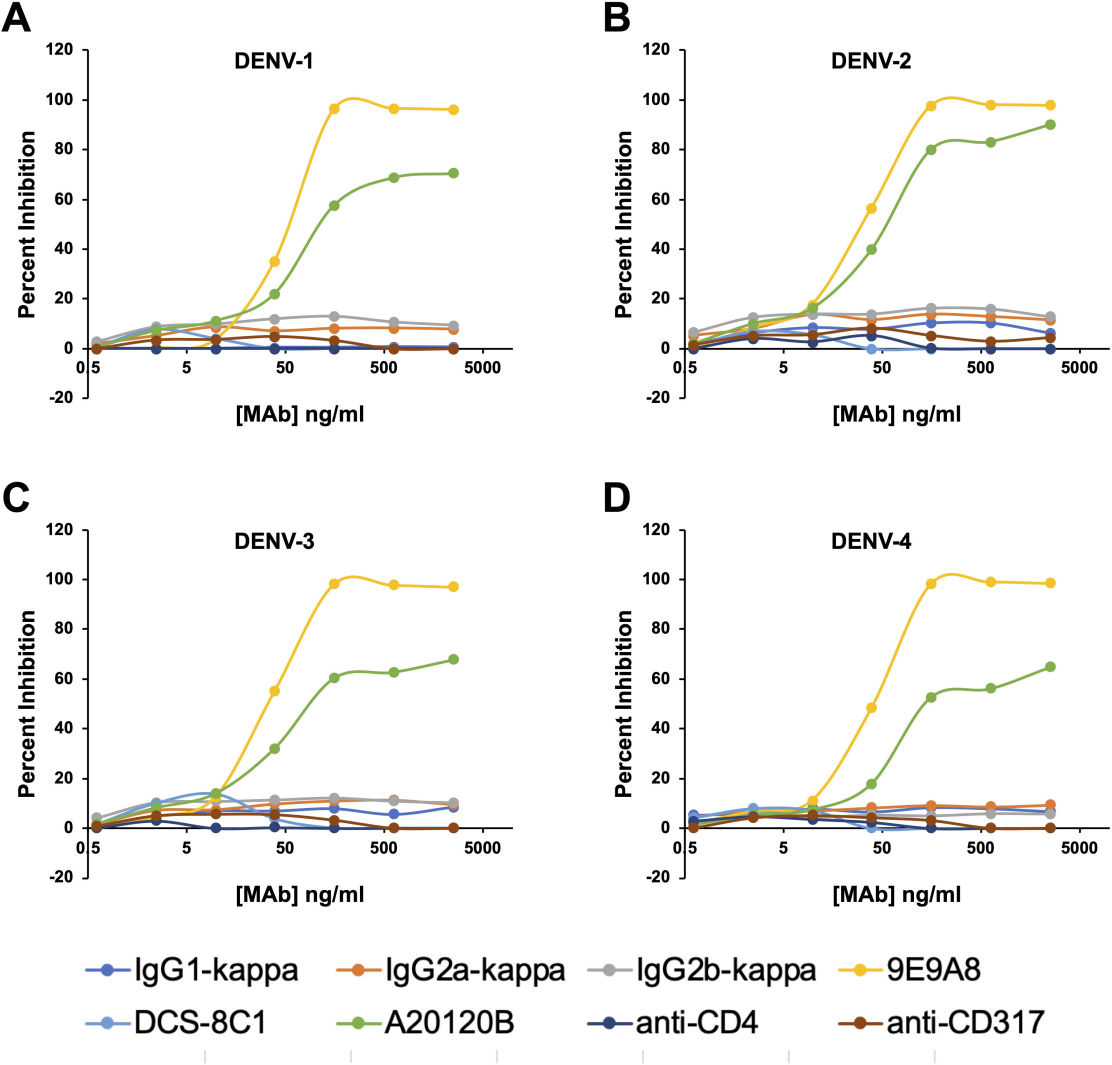
Anti-DC-SIGN MAb viral inhibition curves for all four serotypes on CEM2001 cells (**A–D** for DENV-serotypes 1–4 respectively). Percent inhibition for each of the noted MAbs is shown and was measured at 3 days after culture initiation. These curves were used to calculate the IC_50_ values presented in **Table 1**. Test MAbs were 9A9E8, DCS-8C1 and A20120B, while control MAbs included isotype matched antibodies and two MAbs that bind surface proteins on CEM2001 but should not be involved in viral entry (anti-CD4 and anti-CD317).

We next sought to adapt the culture system to a microwell format to determine whether a short-term culture could be used to measure the direct inhibition of cell-to-cell viral spread. This approach would complement methodologies such PRNT (plaque reduction) and FlowNT (flow cytometry-based neutralization test) neutralization assays. The previously determined replenishment ratio of 20:1 was scaled down accordingly, and a 72-hour incubation period was used to assess the spread of the virus in co-cultures of infected and uninfected CEM2001 cells. To test for direct inhibition of cell-cell virus spread, we used three commercially available DC-SIGN monoclonal antibodies MAbs (clones 9E9A8, A20120B, and DCS-8C1), alongside isotype-matched MAb controls (**Figures 7A–D**, **Supplementary Figure 7**). Among these, 9E9A8 demonstrated a robust inhibitory effect, with an IC_50_ (half-maximal effective concentration) of 33–51 ng/mL for the four serotypes of DENV (**Table 2**). In contrast, A20120B exhibited a measurable IC_50_ but was unable to achieve 100% inhibition for any serotype. Despite having similar binding kinetics and IC_50_ to 9E9A8, DCS-8C1 did not inhibit viral spread (**Table 2**, **Supplementary Figure 7**). Additionally, control MAbs targeting CD4 and CD317 indicated that mere binding of a monoclonal antibody to the cell surface does not non-specifically impede DENV infection spread (**Supplementary Figure 7**). These results collectively confirm that the site specificity of the DC-SIGN MAbs is crucial for their inhibitory function. The effectiveness of 9E9A8 highlights the potential for targeted antibody therapies to block viral spread through specific interactions with host cell receptors, and this adapted microwell assay provides a valuable tool for screening and characterizing the inhibitory potential of MAbs, and potentially small molecule inhibitors, against DENV infection in a high-throughput and scalable format.

**Table 2:** Half-maximum and maximum occupancy and half-maximum virus growth inhibition values for all MAbs tested.

Maximum and Half-maximum occupancy values			Half-maximum virus inhibition values			
MAb	EC_50_	Max. Occupancy	DENV-1	DENV-2	DENV-3	DENV-4
	(ng/ml)	(gMFI)	IC_50_ (ng/ml)			
9E9A8	274	46415	51	33	35	41
DCS-8C1	84	14598	NI	NI	NI	NI
A20120B	3234	19873	175	52	155	336
CD4	286	12102	NI	NI	NI	NI
CD317	619	2270	NI	NI	NI	NI
IgG1𝒌	>10000	86	NI	NI	NI	NI
IgG2a𝒌	>10000	84	NI	NI	NI	NI
IgG2b𝒌	>10000	83	NI	NI	NI	NI

Half-maximum values (IC_50_ and EC_50_) were calculated using an asymmetric, five parameter, non-linear regression curve fit using GraphPad Prism software (version 8.4.3). NI indicates no inhibition observed under the assay conditions.

### Opsonization of a DENV MAb panel on continuous DENV-1–4 infected cell cultures

**Figure 8:**
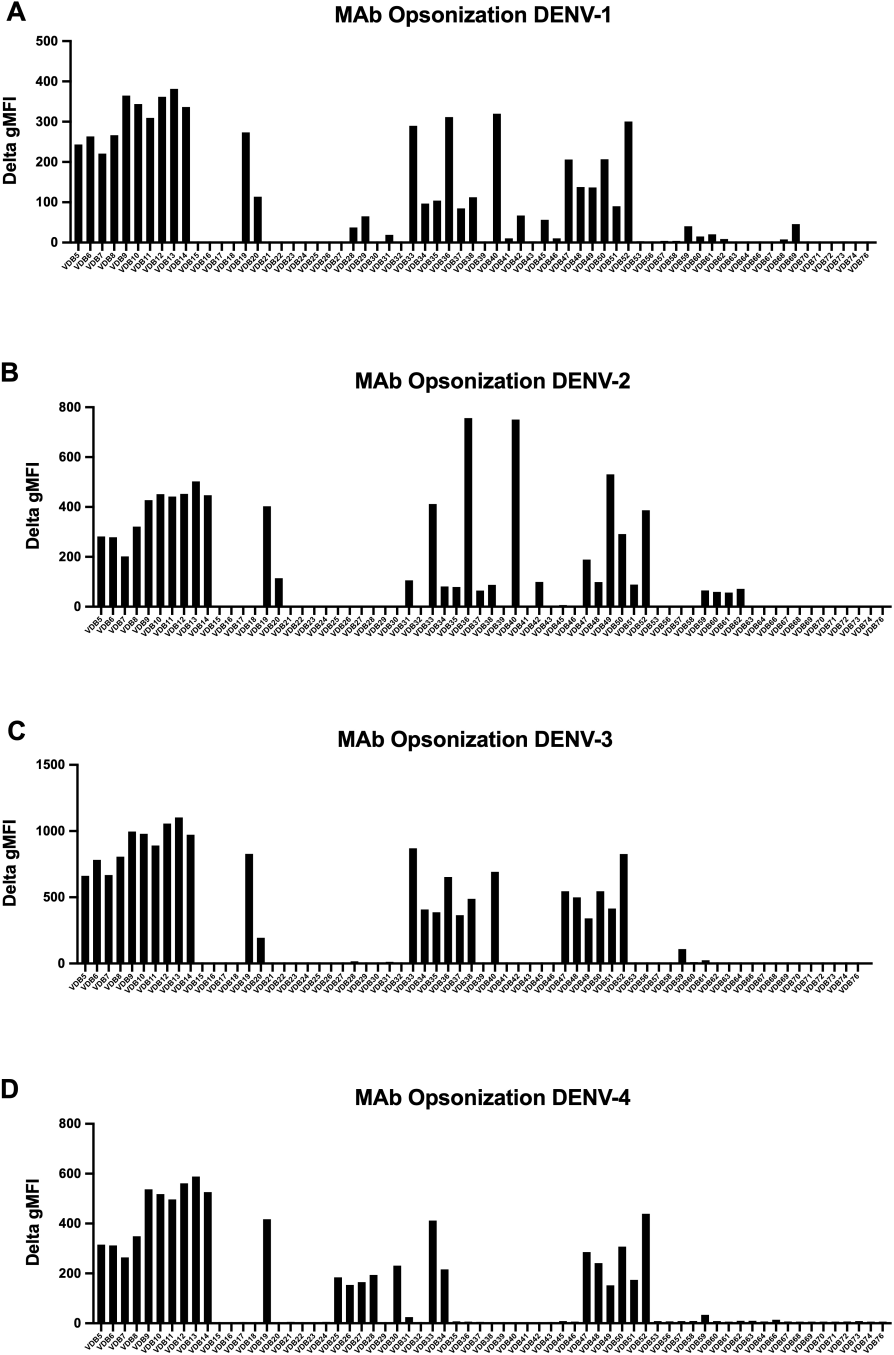
A panel of 67 previously characterized MAbs against DENV were tested for binding to DENV-infected cells. All MAbs were synthesized with a human IgG1 Fc region as previously described (32). Data is shown for infected cells (2H2 positive) for all serotypes **(A–D)** and is presented as the ΔMFI compared to a secondary antibody only control (goat anti-human IgG conjugated to Alexafluor 647). The gating strategy and examples of positive and negative binding MAbs is shown in **Supplementary Figure 8**.

Using a panel of DENV-elicited plasmablast-derived MAbs (67 total) previously generated by our group (32), we proceeded to assess the binding capacity of these E-protein specific MAbs for infected cells. An opsonization assay with the 67 MAb panel (10 µg/mL final) identified those with positive binding across the four DENV serotypes (**Figures 8A–D**, **Supplementary Figure 8**). MAb binding to infected cells (2H2-positive) was quantified as the ΔMFI compared to a secondary only control antibody. Many of the MAbs exhibit multi-serotype binding, presumably to differentially trafficked E protein, which is available for binding on the cell surface (45) (VDB5-14; VDB19; VDB33 and VDB47-52). Several MAbs displayed varying degrees of serotype-specific opsonization. Notably, VDB25 through 28 demonstrate predominantly DENV-4 only binding, whereas VDB36 and VDB40 bind to DENV-1, -2 and -3 but not DENV-4. This data further substantiates the presence of DENV E protein, in addition to NS1 protein, on the surface of infected CEM2001 cells.

### Evaluation of ADCP activity of dengue immune plasma using continuous DENV-1–4 infected cells

**Figure 9:**
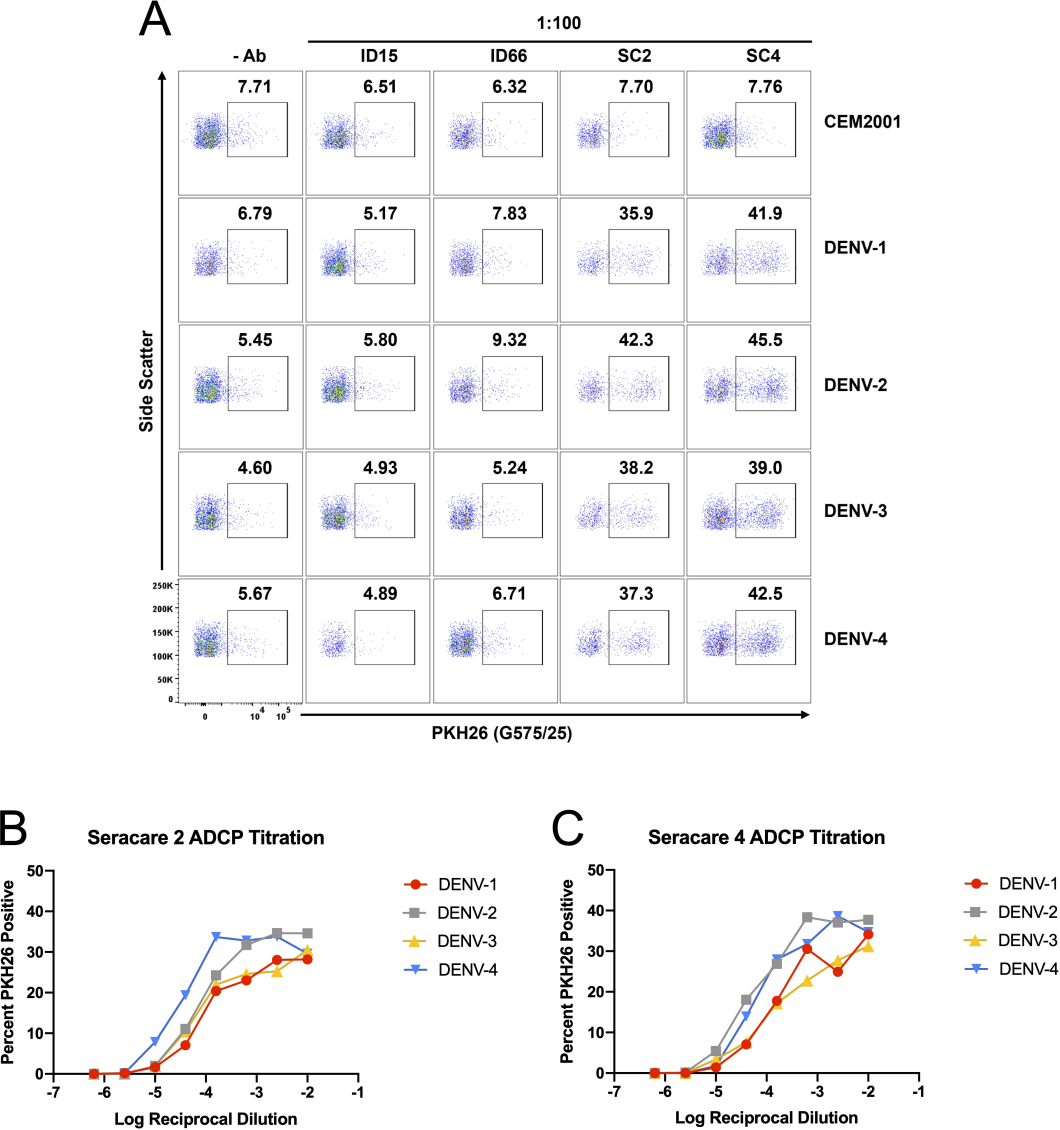
DENV-infected cells are applicable target cells for ADCP assays. **(A)** Plasma (dilued 1:100) from two DENV-naïve subjects (ID15; ID66) and two DENV-immune subjects (SC2; SC4) were compared for ADCP capacity using PKH26 dye staining followed by co-incubation with PBMC-derived CD14^+^ monocytes. Controls include all cells with no antibody source and uninfected CEM2001 cells. A clear signal (s/n = 5.3× to 8.5×) is seen for all four serotypes using DENV-immune plasma. **(B, C)** Titration of the ADCP response was conducted for DENV-plasma and data presented the delta percent PKH26^+^/CD14^+^ monocytes compared with no antibody addition. The gating strategy for CD14^+^ monocytes is shown in **Supplementary Figure 9**.

To further leverage the continuous DENV culture system, we conducted an ADCP assay to assess the phagocytic activity of dengue immune plasma against all four DENV serotypes. ADCP involves phagocytes such as monocytes, macrophages, dendritic cells, or neutrophils engaging via their Fcγ receptors, target cells coated with antibodies, resulting in the internalization and degradation of the target cells (17). In this assay, target cells (infected CEM2001 cells) were first labeled with the cell membrane dye PKH26 (**Supplementary Figure 9A**), then opsonized with plasma. These opsonized target cells were subsequently co-incubated with cryopreserved PBMCs from healthy donors. After one hour, PKH26^+^/CD14^+^ monocytes were measured via flow cytometry to determine the percentage of phagocytosis. **Figure 9A** shows the ADCP response to plasma from two DENV-immune donors (SeraCare Proficiency Panel Plasma SC2 and SC4) and two DENV-naïve subjects. At a plasma dilution of 1:100 the DENV-immune plasma shows a clear increase in PKH26^+^ monocytes compared with DENV-naïve plasma. Furthermore, plasma from both subjects demonstrated ADCP activity in a dose-dependent, antibody-mediated effect across all DENV serotypes for dengue-immune plasma relative to dengue naïve plasma. SC4 exhibited moderately higher phagocytic responses compared to SC2. (**Figures 9B, C**). Additionally, both subjects showed higher ADCP levels for DENV-2 and DENV-4, highlighting a potential serotype-specific immunity possibly indicative of prior serotype infection history. These results emphasize the individual variability in antibody responses, with implications for developing and evaluating therapeutic antibodies and vaccine efficacy.

Subsequently, we performed the ADCP assay as above using the 67 MAb panel from the opsonization experiment (see **Figure 8**) to further assess function. Although the overall ADCP activity was lower than that induced by dengue-immune plasma (**Supplementary Figure 9B**), this result is expected since polyclonal antibodies can target multiple sites at different angles on a target antigen, which can then cluster FcRs and trigger more efficient uptake by phagocytic cells (48). Notably, the DENV-1 ADCP results paralleled those of the opsonization assay for certain VDB MAbs (5, 11, 12, 13, 19, 33, 36, 40 and 50; **Figures 8A–D**), however significant serotype-specific functionality was observed. This serotype specificity is particularly evident for DENV-1 compared to DENV-2-4, possibly a result of immune imprinting from the MAbs, which were derived from individuals with a primary DENV-1 infection followed by a secondary DENV-1 or DENV-3 infection (32). These results illustrate that while MAbs may exhibit broad binding across the four serotypes, their functional activity can be more serotype-specific, underlining the complex interplay of antibody binding and functional outcome in DENV immune responses.

### Evaluation of ADCD activity of dengue immune plasma using continuous DENV-1–4 infected cells

**Figure 10:**
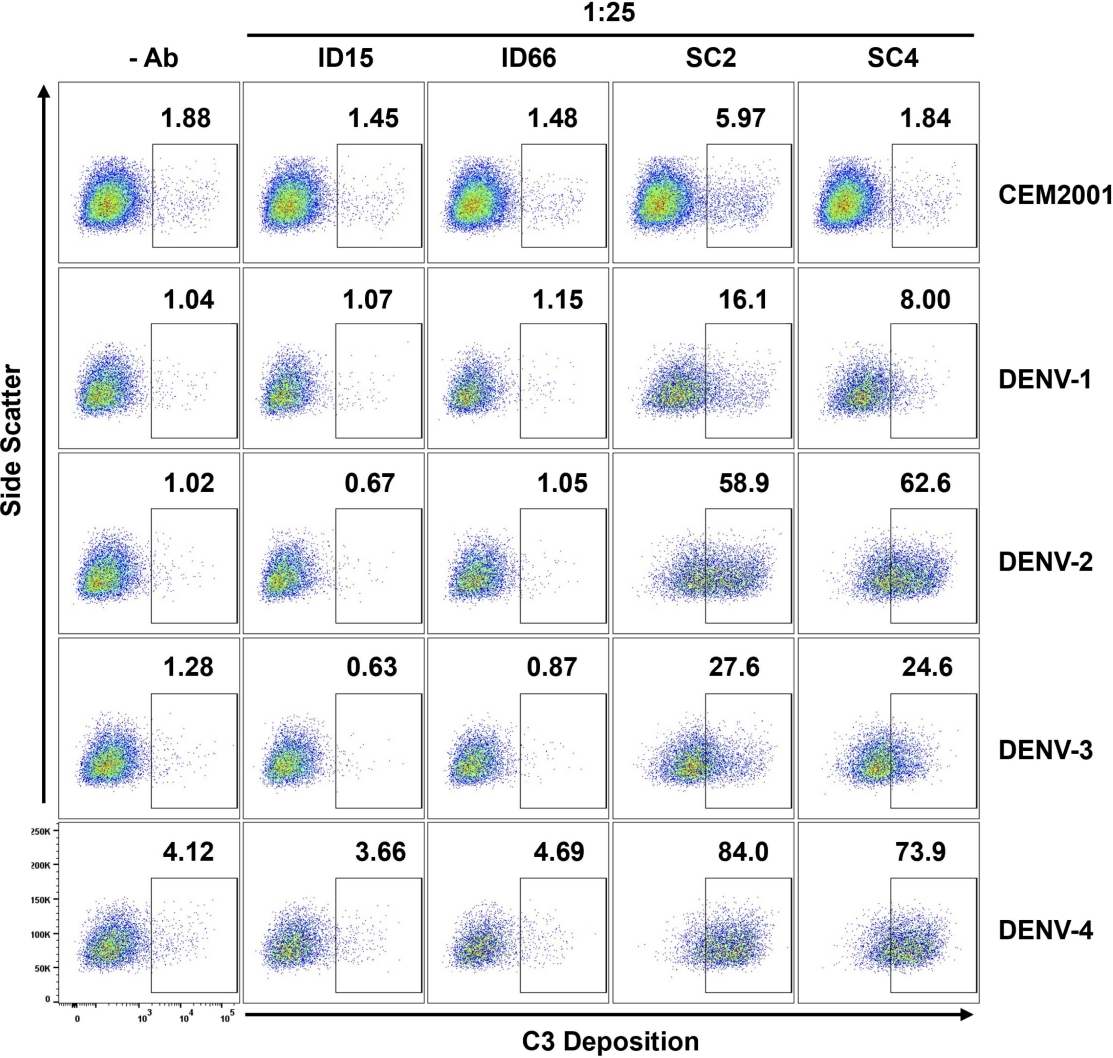
DENV-infected cells (2H2^+^) are suitable target cells for ADCD assays. Plasma (diluted 1:25) from two DENV-naïve subjects (ID15; ID66) and two DENV-immune subjects (SC2; SC4) were compared for ADCD activity using plasma antibody-opsonized infected target cells co-incubated with Low-Tox New Guinea Pig complement. C3 deposition was detected using FITC-conjugated goat anti–Guinea Pig Complement C3 antibody and analyzed by flow cytometry. Controls include all cells with no antibody source and uninfected CEM2001 cells. The gating strategy for DENV-infected target cells showing C3 deposition and ADCD activity using DENV-immune plasma is shown in **Supplementary Figure 10A**. Comparative analysis of ADCD activity on DENV-uninfected cells (2H2^-^) from the same plasma and same experiment is shown in **Supplementary Figure 10B**.

Finally, we evaluated ADCD using the same DENV-immune and DENV-naïve plasma samples described in the ADCP assay. At a 1:25 dilution, plasma from DENV-immune donors induced significantly higher levels of complement C3 deposition on infected target cells compared to plasma from DENV-naïve individuals. Consistent with the ADCP results, this enhanced ADCD activity was most notable against DENV-2 and DENV-4 infected cells (**Figure 10**, **Supplementary Figure 10**). These findings suggest that antibodies elicited during natural infection can mediate distinct but complementary effector functions. Together, these results emphasize the importance of serotype-specific, multifunctional antibody responses in shaping protective immunity and informing therapeutic development for dengue.

## Discussion

This study establishes a continuous DENV infection model in CEM2001 cells that supports all four serotypes, overcoming the limitations of traditional MOI-based methods that require repeated infections and limit scalability. By periodically replenishing uninfected cells, we sustain stable infections suitable for high-throughput and long-term assays, improving consistency and reducing logistical burden. The utility of the system presented here is predicated on the ability of virus-infected cells to express surface viral antigen bound and opsonized by antibodies in the serum of previously DENV-infected individuals, a critical feature for evaluating immune responses elicited by infection or vaccination. Altogether, this platform enables robust functional characterization of serotype-specific and cross-reactive antibodies across all four serotypes.

Maintaining long-term infection reduces experimental variability and enables high-throughput screening of antibody responses and antiviral candidates. The CEM-NK^R^ T-lymphoblastoid cell line was chosen because it does not express appreciable levels of common Fc receptors, is resistant to direct lysis by human NK cells, and does not activate human monocytes. These traits make it well suited for measuring antibody effector functions in isolation from FcR-dependent confounders. The use of the continuous infection system to assess antibody responses through various immunoassays—such as ADCP and ADCD assays— enables dissection of antibody function beyond neutralization.

The robust inhibitory effect of the DC-SIGN-specific MAb 9E9A8, as demonstrated in our microwell assay, underscores the potential of this system for screening targeted inhibitors of viral spread. While this readout reflects inhibition of cell-to-cell viral transmission rather than classical neutralization of cell-free virus, it provides a biologically relevant complement to assays such as PRNT or FlowNT. We have therefore positioned this system as an orthogonal tool for functional antibody characterization that complements, rather than replaces, traditional neutralization assays such as PRNT. Its reproducibility—demonstrated by consistent infection kinetics and cell expansion across ten serial passages (**Figure 6**)—further supports its utility as a stable and scalable platform. Benchmarking studies using standardized controls and reference materials will be important to facilitate cross-platform comparison and validation.

Evaluation of plasmablast-derived DENV MAbs highlights the complexity of the antibody response. While the MAbs displayed broad binding across serotypes, functional activity remained highly serotype-specific, stressing the importance of considering both binding and effector functions when assessing the antibody response to potential vaccine candidates. Fc-mediated functions such as ADCP and ADCD—assessed using this system—have been associated with protection against dengue in both naturally infected and vaccinated individuals (25, 29) and are broadly supported by studies across infectious diseases (17). In the flavivirus field specifically, murine models of Yellow Fever, West Nile, and Zika virus have demonstrated that antibody-mediated protection can depend critically on Fc effector functions, including complement activation and FcγR engagement (49–52). While there has been increasing attention to cell-mediated immunity in dengue, including efforts to elicit robust T cell responses through vaccine design (*e.g.*, Qdenga) (53–59), systematic evaluation of antibody Fc-effector functions remains limited. This represents a significant gap given the central role antibodies play in both protection and pathogenesis and underscores the need for scalable tools to characterize antibody effector function across dengue serotypes.

The serotype specificity observed in ADCP activity here, particularly for DENV-1, suggests that other qualities of the antibody response such as isotype, glycosylation state or fine specificity determine the functional capacity (9). While our evaluation employed a panel of infection-derived MAbs with defined serotype profiles, incorporating commercial serotype-specific antibodies in future studies could provide standardized reference points for antibody binding and function, facilitating cross-study comparisons. These findings stress the need for vaccine strategies that elicit broad and functionally effective antibody responses while minimizing the risk of ADE—an important goal in light of the mixed clinical outcomes of current dengue vaccines (60). Other Fc-mediated effector functions, such as ADCC, may also contribute to protection or pathogenesis in dengue and could be evaluated using this system in future studies. Although the current FcR-null platform is not designed to model ADE, future adaptations incorporating FcR^+^ target cells or co-culture models may enable investigation of ADE mechanisms during heterotypic DENV infection. A better understanding of the relationship between antibody binding and downstream functional activity will support the rational design of vaccines providing durable, cross-serotype protection.

Our continuous infection cell culture system offers a versatile platform with potential applications beyond DENV. Viruses such as HIV-1, Ebola, Marburg, SARS-CoV, and Zika also utilize DC-SIGN for cell entry (61–67), suggesting that the CEM2001 cell line may serve as a model for investigating pathogenesis and host–virus interactions across multiple pathogens. By supporting sustained, serotype-inclusive DENV infection through DC-SIGN rather than FcR pathways, our system enables controlled, scalable studies while avoiding receptor-related variability seen in traditional FcR+ cell lines like K562 or U937 (68–70).

As with any model system, there are limitations. Variability in DC-SIGN expression and the use of a single receptor may affect consistency and fail to fully reflect the complexity of *in vivo* infections. Additionally, the use of a monoclonal T cell line may not capture tissue-specific differences in host cell interactions. Genetic drift over prolonged culture could also impact reproducibility. Future studies incorporating primary human cells or co-culture systems may improve physiological relevance and model refinement.

Together, these findings establish a stable and scalable model for functional antibody studies across all four DENV serotypes. Integrating this system with co-culture platforms, single-cell technologies, and advanced imaging may further expand its utility in dissecting virus–host dynamics. Its compatibility with high-throughput immunoassays and potential adaptability for ADE modeling position it as a valuable tool for characterizing both protective and pathogenic antibody responses. Refining and leveraging this model could support the development of more effective vaccines, therapeutic antibodies, and strategies for combating dengue and other emerging viral threats.

## Data Availability

The raw data supporting the conclusions of this article will be made available by the authors, without undue reservation.
