# Supplementary material for: Applications and insights from continuous dengue virus infection in a stable cell line
Source: Front Immunol. 2025 Jun 24;16:1618650. doi: 10.3389/fimmu.2025.1618650 (PMC12234473; doi:10.3389/fimmu.2025.1618650)

Supplementary Figure 1: The CEM1 clone was obtained by limiting dilution cloning of the parent cell line CEM-NK^R. CEM1 cells were subsequently transfected with an expression plasmid (pcDNA3.1+) carrying a human DC-SIGN (CD209 transcript variant 1: NM_021155) insert with a 5' Kozak sequence appended (**A**). After selection (**B**), enrichment sorting and cloning (**C**), expanded clones were screened for uniform, stable surface expression of human DC-SIGN. The clone selected for stability testing and characterization was designated CEM2001 (**D**). CEM2001 displayed stable high-level CD209 expression through 4 passages (freeze-thaw cycles) and as expected, demonstrated permissivity to infection with all four serotypes of DENV (see Figure 1, panel C).

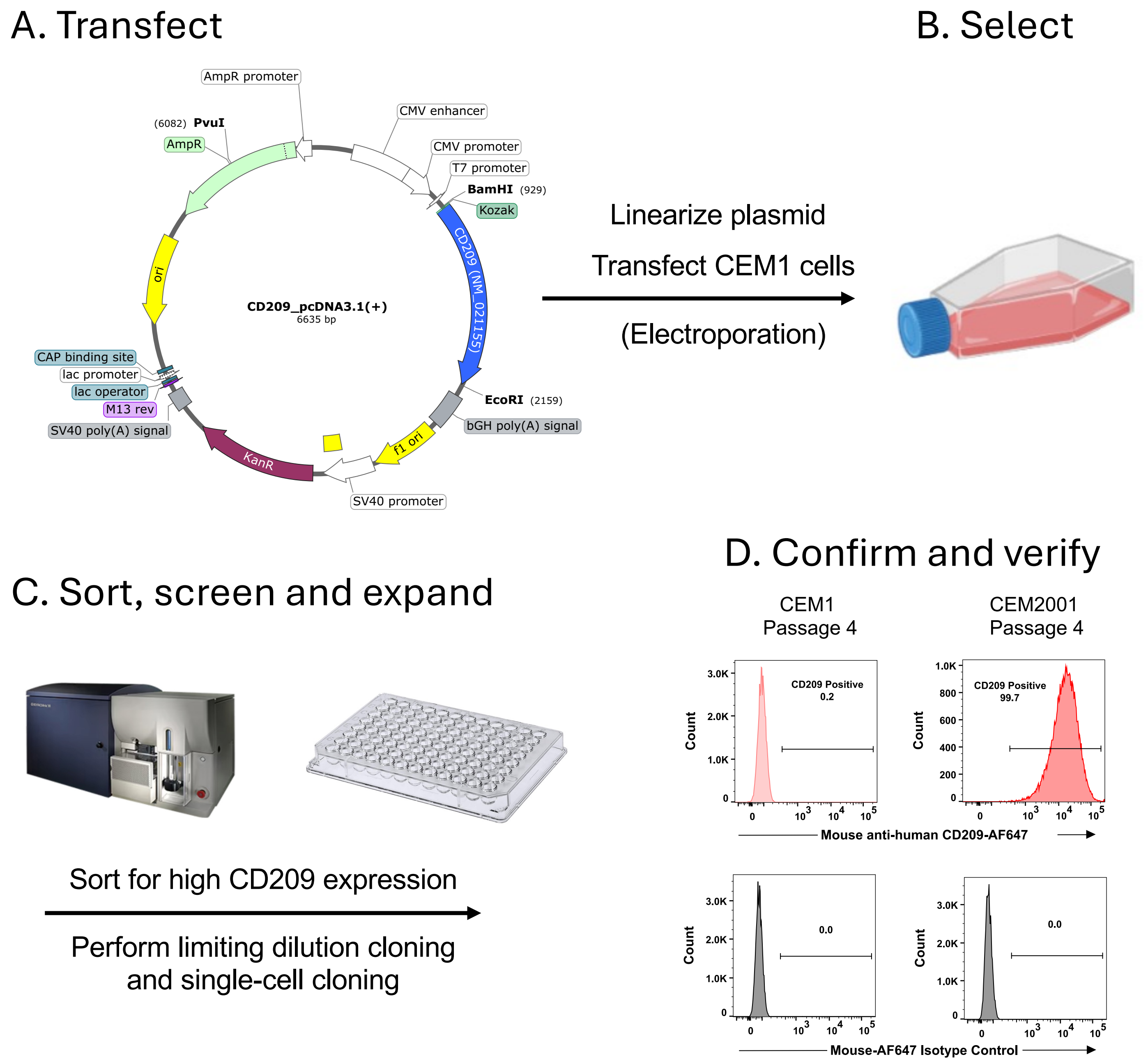

Supplement: Supplementary file 1 [file DataSheet1.pdf]
