# Supplementary material for: Applications and insights from continuous dengue virus infection in a stable cell line
Source: Front Immunol. 2025 Jun 24;16:1618650. doi: 10.3389/fimmu.2025.1618650 (PMC12234473; doi:10.3389/fimmu.2025.1618650)
Supplement: Supplementary file 2 [file DataSheet2.pdf]

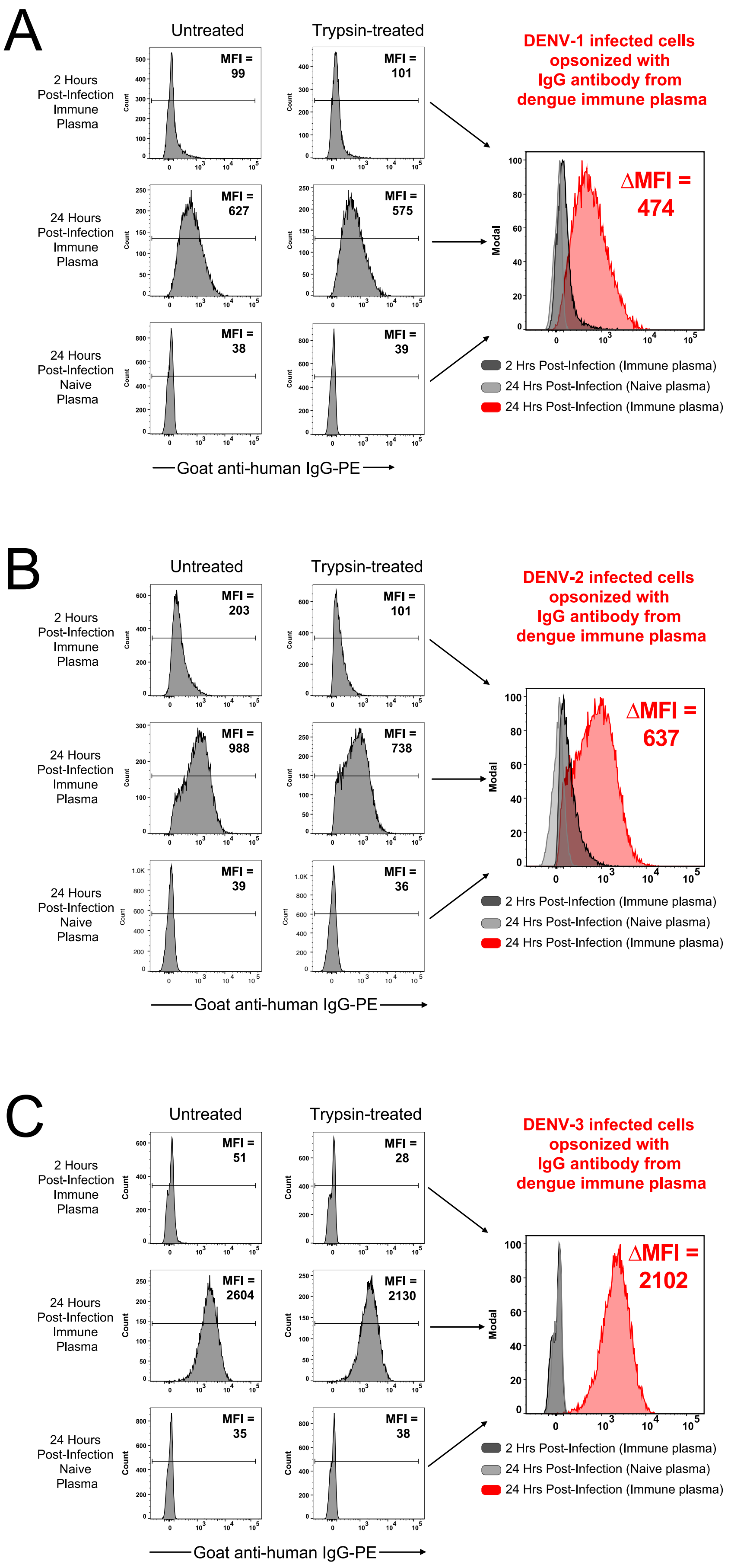

Supplementary Figure 2: Panels **A, B** and **C** show the same gating strategy as shown in Figure 2, panel B (DENV-4) is shown for DENV-1, DENV-2 and DENV-3, respectively. Variability in the binding of virus particles to the surface of CEM2001 cells is evident for the different serotype preparations. However, trypsin treatment at 2 hours post-infection reduces binding signal to negligible levels. A clear increase in *de novo* surface antigen expression post-infection can be seen by 24 hours for all serotypes.
