# Supplementary material for: Applications and insights from continuous dengue virus infection in a stable cell line
Source: Front Immunol. 2025 Jun 24;16:1618650. doi: 10.3389/fimmu.2025.1618650 (PMC12234473; doi:10.3389/fimmu.2025.1618650)
Supplement: Supplementary file 3 [file DataSheet3.pdf]

<u>Supplementary Figure 3</u>: Panels **A, B, C** and **D** show expanded gating strategies for all four serotypes shown in Figure 3. Included for reference are non-infected cultures of CEM2001 and staining with dengue-naïve plasma to demonstrate antibody specificity for surface binding of IgG from dengue immune plasma on only infected cells (2H2-poistive).

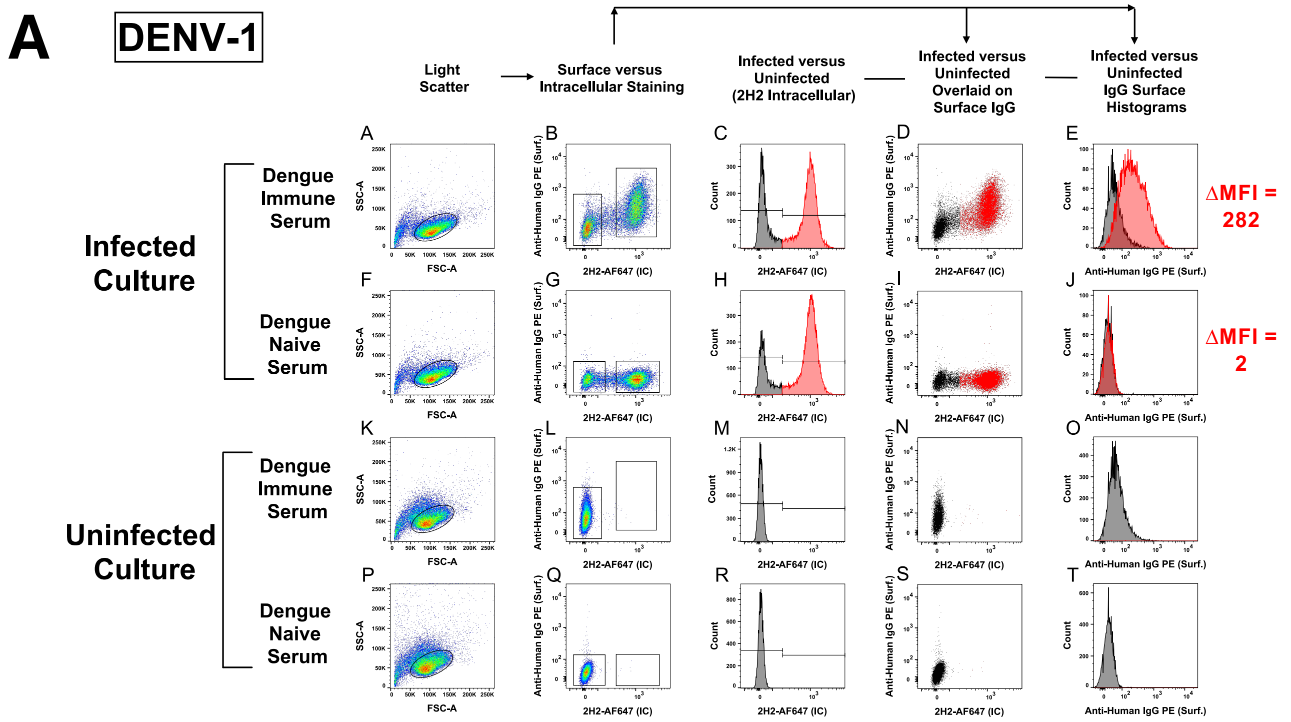

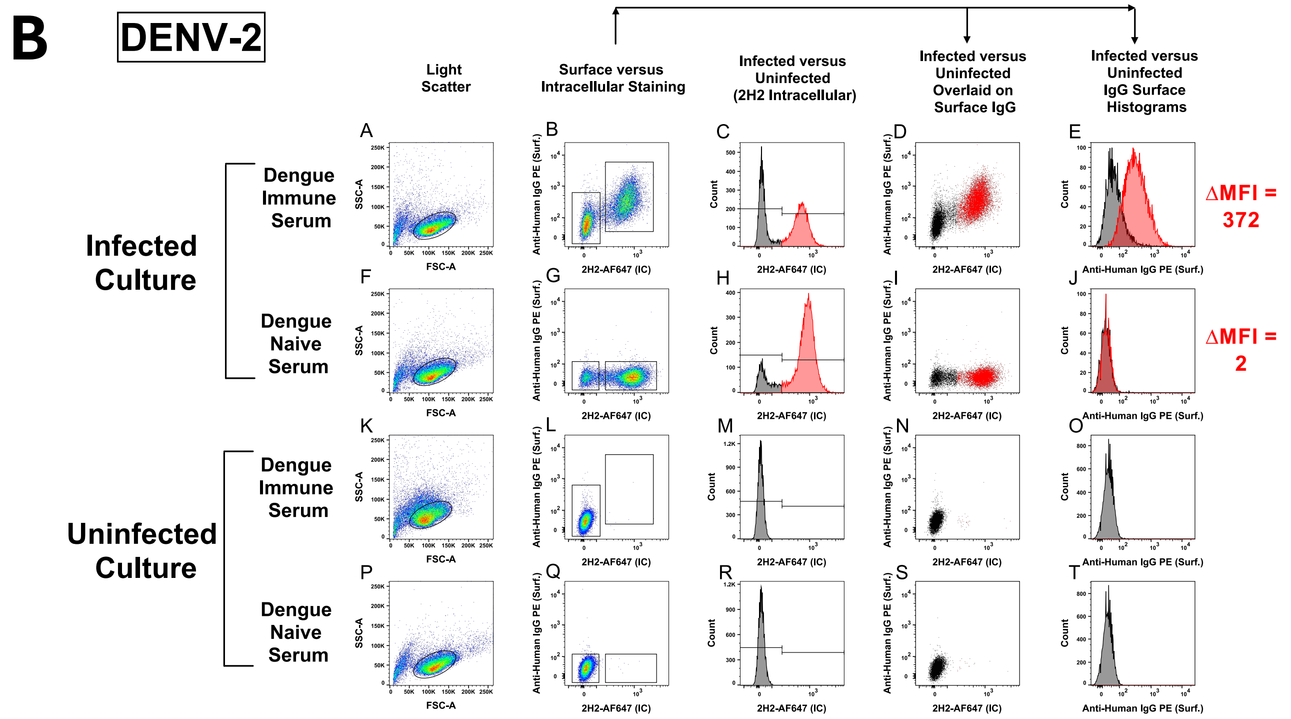

C

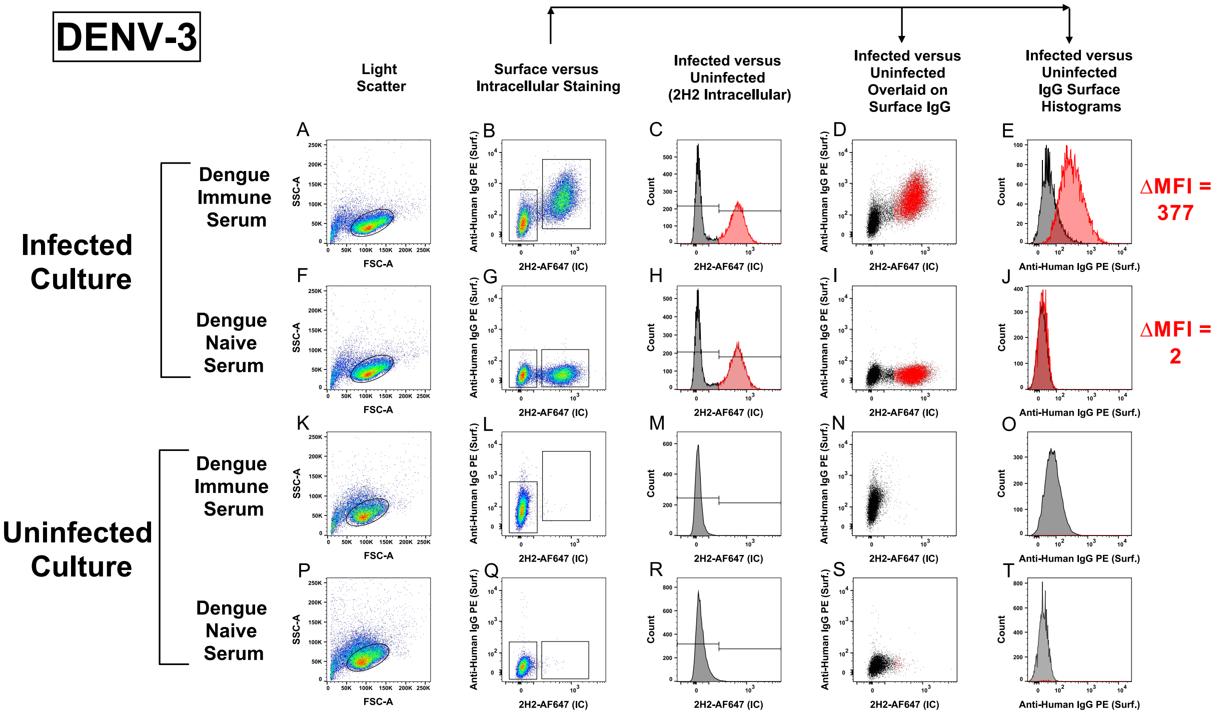

DENV-3
Light Scatter
Surface versus Intracellular Staining
Infected versus Uninfected (2H2 Intracellular)
Infected versus Uninfected Overlaid on Surface IgG
Infected versus Uninfected IgG Surface Histograms
Infected Culture
Dengue Immune Serum
Dengue Naive Serum
Uninfected Culture
Dengue Immune Serum
Dengue Naive Serum
A
B
C
D
E
F
G
H
I
J
K
L
M
N
O
P
Q
R
S
T
SSC-A
FSC-A
Anti-Human IgG PE (Surf.)
2H2-AF647 (IC)
Count
Anti-Human IgG PE (Surf.)
ΔMFI = 377
ΔMFI = 2

D

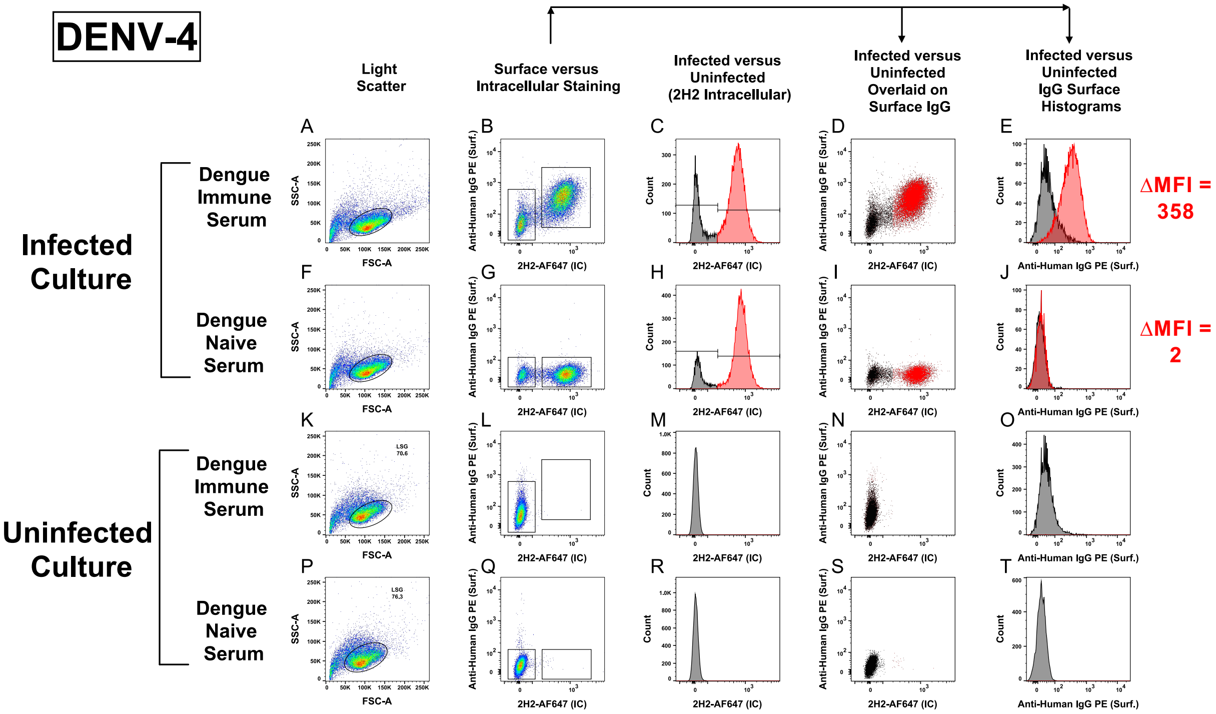

DENV-4
Light Scatter
Surface versus Intracellular Staining
Infected versus Uninfected (2H2 Intracellular)
Infected versus Uninfected Overlaid on Surface IgG
Infected versus Uninfected IgG Surface Histograms
Infected Culture
Dengue Immune Serum
Dengue Naive Serum
Uninfected Culture
Dengue Immune Serum
Dengue Naive Serum
A
B
C
D
E
F
G
H
I
J
K
L
M
N
O
P
Q
R
S
T
SSC-A
FSC-A
Anti-Human IgG PE (Surf.)
2H2-AF647 (IC)
Count
Anti-Human IgG PE (Surf.)
ΔMFI = 358
ΔMFI = 2
