# Supplementary material for: Applications and insights from continuous dengue virus infection in a stable cell line
Source: Front Immunol. 2025 Jun 24;16:1618650. doi: 10.3389/fimmu.2025.1618650 (PMC12234473; doi:10.3389/fimmu.2025.1618650)

Supplementary Figure 4: Intracellular 2H2 expression exceeds surface expression and identifies infected cells. CEM2001 were infected with serial dilutions of all four serotypes of DENV and were assessed for intracellular and surface expression of 2H2 at an MOI between 0.5 and 0.75. Cells were first surface stained with a saturating concentration of 2H2-FITC conjugate, then fixed, permeabilized and stained intracellularly with a 2H2-APC conjugate. Data is presented as follows: (**A**) Dot-plots of all cells from the light scatter gate showing 2H2-FITC (surface) versus 2H2-APC (intracellular); (**B**) Overlap dot-plot of each infected cell culture with the uninfected cell culture; (**C**) overlay of 2H2-APC (intracellular) histograms for each infected cell culture with the uninfected cell culture; (**D**) overlay of 2H2-FITC (surface) histograms for each infected cell culture with the uninfected cell culture. The frequency of positive cells in each gate is denoted.

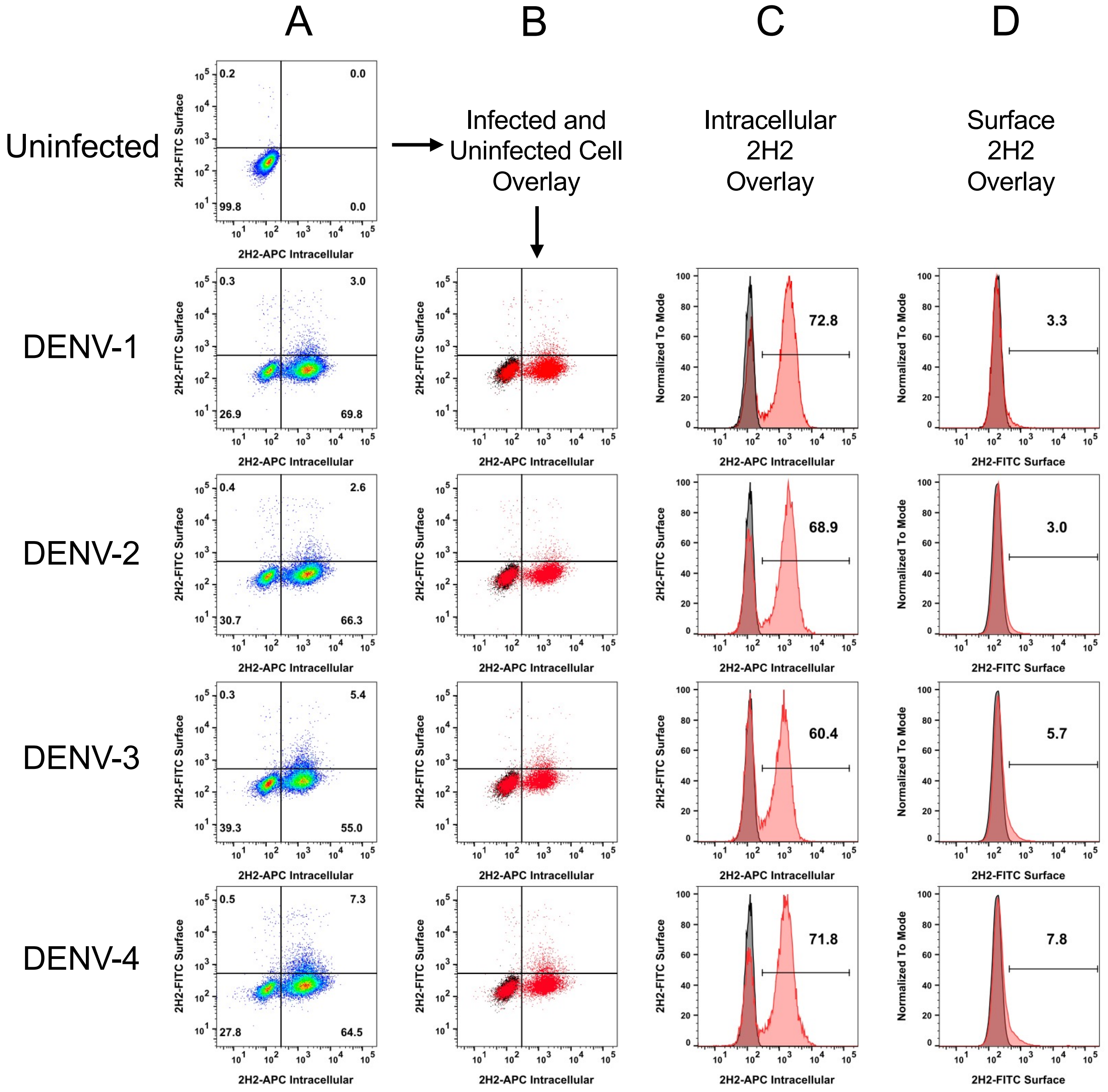

Supplement: Supplementary file 4 [file DataSheet4.pdf]
