# Supplementary material for: Applications and insights from continuous dengue virus infection in a stable cell line
Source: Front Immunol. 2025 Jun 24;16:1618650. doi: 10.3389/fimmu.2025.1618650 (PMC12234473; doi:10.3389/fimmu.2025.1618650)
Supplement: Supplementary file 5 [file DataSheet5.pdf]

Supplementary Figure 5: Add back ratios of 4:1 to 20:1 result in similar levels of virus infected cells three days post-replenishment. Replenishment of infected cultures with uninfected CEM2001 was performed (set up day) and the percent of infected cells in set up cultures compared with 3 days of culture. All four serotypes demonstrate comparable and rapid spread of virus in a 3 day culture period.

Percent infected cells at culture set up and 3 days post-replenishment*

Add back ratio		DENV-1	DENV-2	DENV-3	DENV-4
4:1	Set up	2.4	2.0	1.1	2.3
	Day 3	65.1	50.2	67.3	61.6
10:1	Set up	1.1	0.9	0.5	1.1
	Day 3	70.6	52.7	69.1	69.4
20:1	Set up	0.6	0.5	0.3	0.6
	Day 3	69.6	45.1	64.0	69.8

* Uninfected CEM2001 cells = 0.2% (2H2 positive)
