# Supplementary material for: Applications and insights from continuous dengue virus infection in a stable cell line
Source: Front Immunol. 2025 Jun 24;16:1618650. doi: 10.3389/fimmu.2025.1618650 (PMC12234473; doi:10.3389/fimmu.2025.1618650)

Supplementary Figure 6: Passage 10 replenishment cultures retain DENV-immune plasma binding capacity. Level of infection (intracellular 2H2 expression) and ability to maintain equivalent opsonizing antibody binding was monitored in CEM2001 cells infected with all four serotypes of DENV through 10 passages. Each passage involved 1 week of culture with replenishment of uninfected cells (see Methods and Materials and Results Figure 6). Gating strategy depicts viable cells by light scatter, then assessed for intracellular 2H2 and finally an overlay of infected and uninfected cells surface stained with DENV-immune plasma or DENV-naïve plasma and stained with goat anti-human IgG polyclonal antibody (AF647 conjugated).

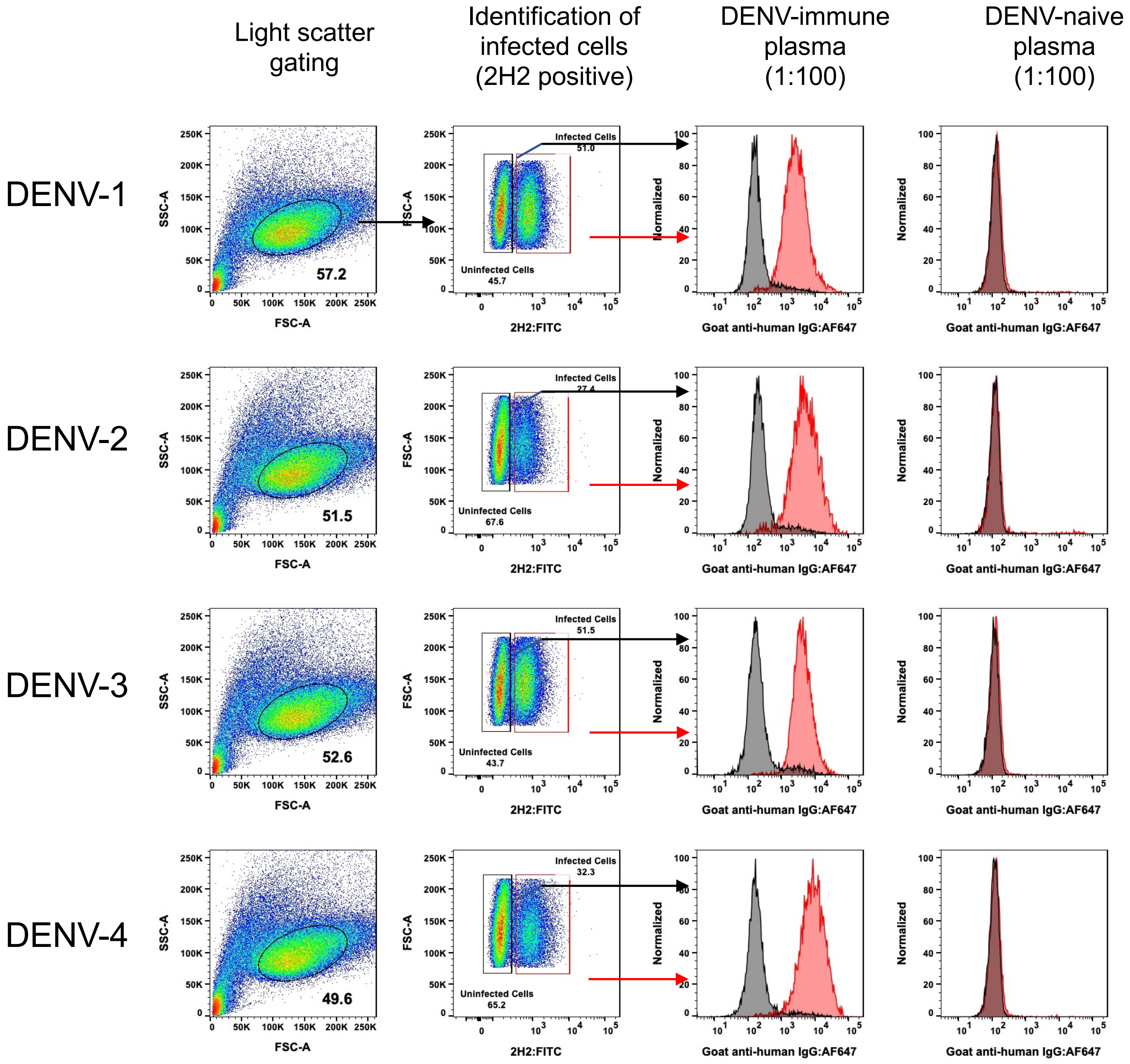

Supplement: Supplementary file 6 [file DataSheet6.pdf]
