# Supplementary material for: Applications and insights from continuous dengue virus infection in a stable cell line
Source: Front Immunol. 2025 Jun 24;16:1618650. doi: 10.3389/fimmu.2025.1618650 (PMC12234473; doi:10.3389/fimmu.2025.1618650)
Supplement: Supplementary file 7 [file DataSheet7.pdf]

Supplementary Figure 7: Panel **A** shows binding curve titrations on CEM2001 cells for each of the antibodies used in the inhibition experiment (Figure 7). None of the three control MAbs (isotypes) bind to CEM2001 cells while all five test MAbs present discreet binding curves. Importantly anti-CD4 and anti-CD317 bind to the target cells, but do not inhibit virus infection further confirming specificity of the inhibitory effect of MAbs 9E9A8 and A201120B. The calculated EC_{50} and maximum occupancy values are shown Table 2 (main text). Notably, DCS-8C1 has the lowest EC_{50} yet does not inhibit virus infection to any appreciable level, while the best inhibitory antibody, 9E9A8, shows the highest maximal occupancy level. When a concentration of MAb was selected which resulted in equivalent surface occupancy of DC-SIGN on CEM2001, panel **B**, the inhibition of virus infection was compared (panel **C**). While the occupancy level of a MAb is clearly important for determining the IC_{50} of inhibition, the binding site of these three MAbs is clearly different and is the most important determinant of the inhibitory effect.

A

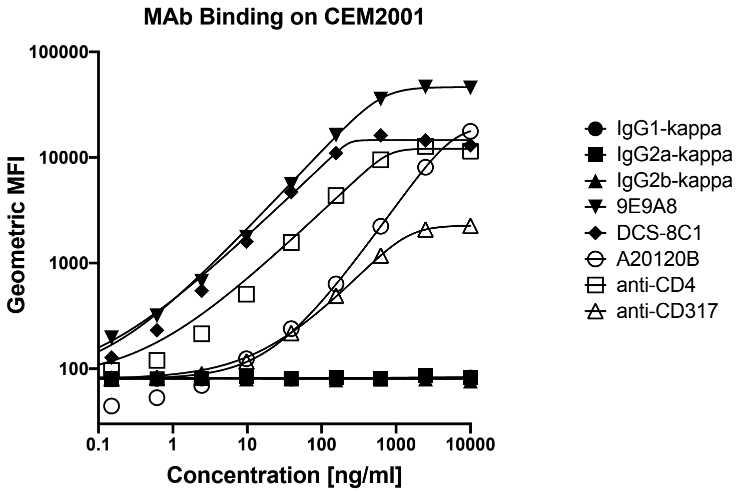

B

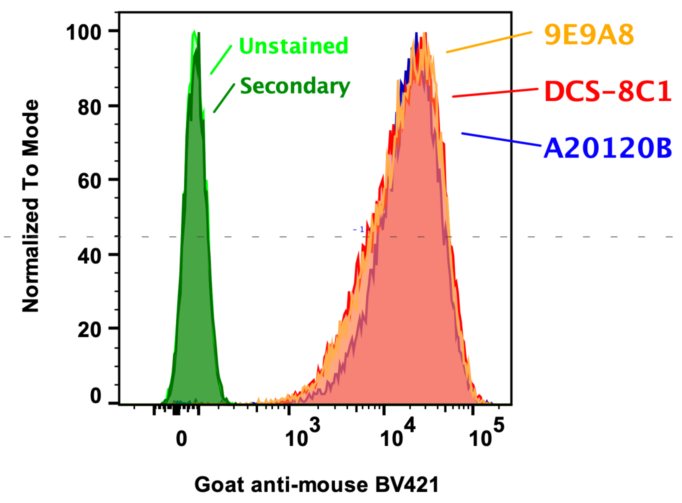

C

MAb		9E9A8	DCS-8C1	A20120B
[MAb]	(ng/ml)	156	625	10000
CEM2001	(gMFI)	16269	16204	17716
CEM1 (Parent)		85	85	86
DENV-1	% Inhibition	96.4	0	70.5
DENV-2		97.7	0	90.0
DENV-3		98.0	0	67.7
DENV-4		98.2	0	64.7
