# Supplementary material for: Applications and insights from continuous dengue virus infection in a stable cell line
Source: Front Immunol. 2025 Jun 24;16:1618650. doi: 10.3389/fimmu.2025.1618650 (PMC12234473; doi:10.3389/fimmu.2025.1618650)

Supplementary Figure 8: Replenishment cultures of CEM2001 infected with all four serotypes of DENV were tested for their ability to bind MAbs with known specificity for DENV E protein. Panel **A** shows control opsonization with negative and positive control plasma on un-infected and infected cells (defined by intracellular 2H2 expression) and subsequently surface stained with goat anti-human IgG polyclonal antibody (AF647 conjugated). All MAbs tested were synthesized and expressed with a human IgG1 constant region (see Methods and Materials). **B**. An example of two MAbs with different binding capacity for surface DENV E protein. VDB21 does not bind DENV-3 infected cells, while VDB13 clearly binds DENV-3 infected cells. Furthermore, VDB13 shows binding to all four serotypes of DENV.

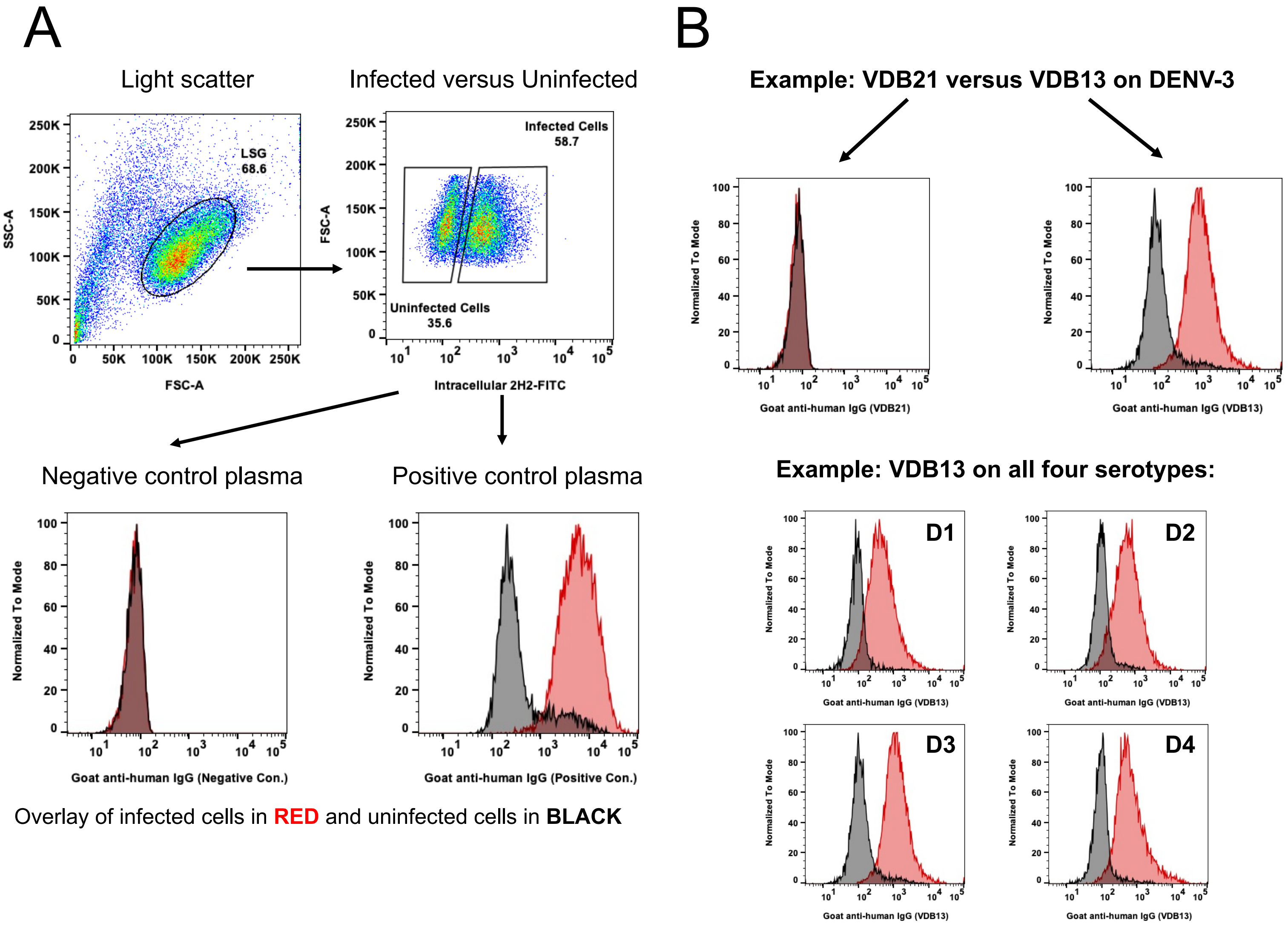

Supplement: Supplementary file 8 [file DataSheet8.pdf]
