# Supplementary material for: Applications and insights from continuous dengue virus infection in a stable cell line
Source: Front Immunol. 2025 Jun 24;16:1618650. doi: 10.3389/fimmu.2025.1618650 (PMC12234473; doi:10.3389/fimmu.2025.1618650)
Supplement: Supplementary file 9 [file DataSheet9.pdf]

Supplementary Figure 9: **A.** Gating strategy (below) for the ADCP assay and determination of PKH26 uptake by $CD14^{+}$ monocytes within PBMCs. Co-incubation of PBMC with PKH26 loaded CEM2001 was performed and the subsequent uptake of PKH26 by the $CD14^{+}$ monocyte population is shown. **B.** A panel of 67 previously characterized MAbs against DENV were tested for ADCP activity using infected cells for all four serotypes. Data is as $PKH26^{+}$ positive monocytes with background subtracted. Gating strategy was identical to that presented in panel A.

A

Gating Strategy 1 – CEM2001 + donor 10, no Ab

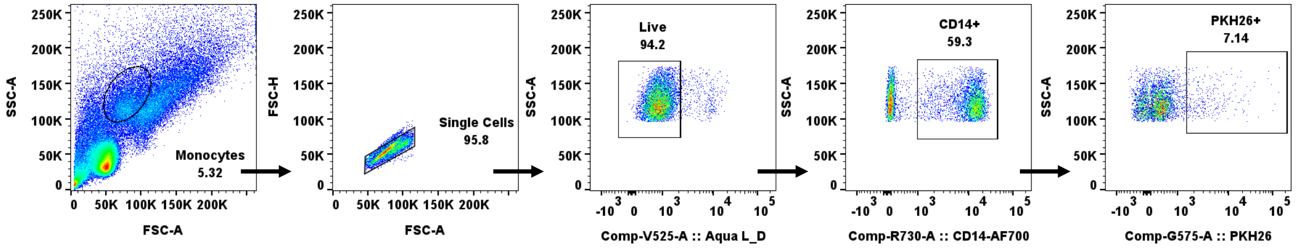

B

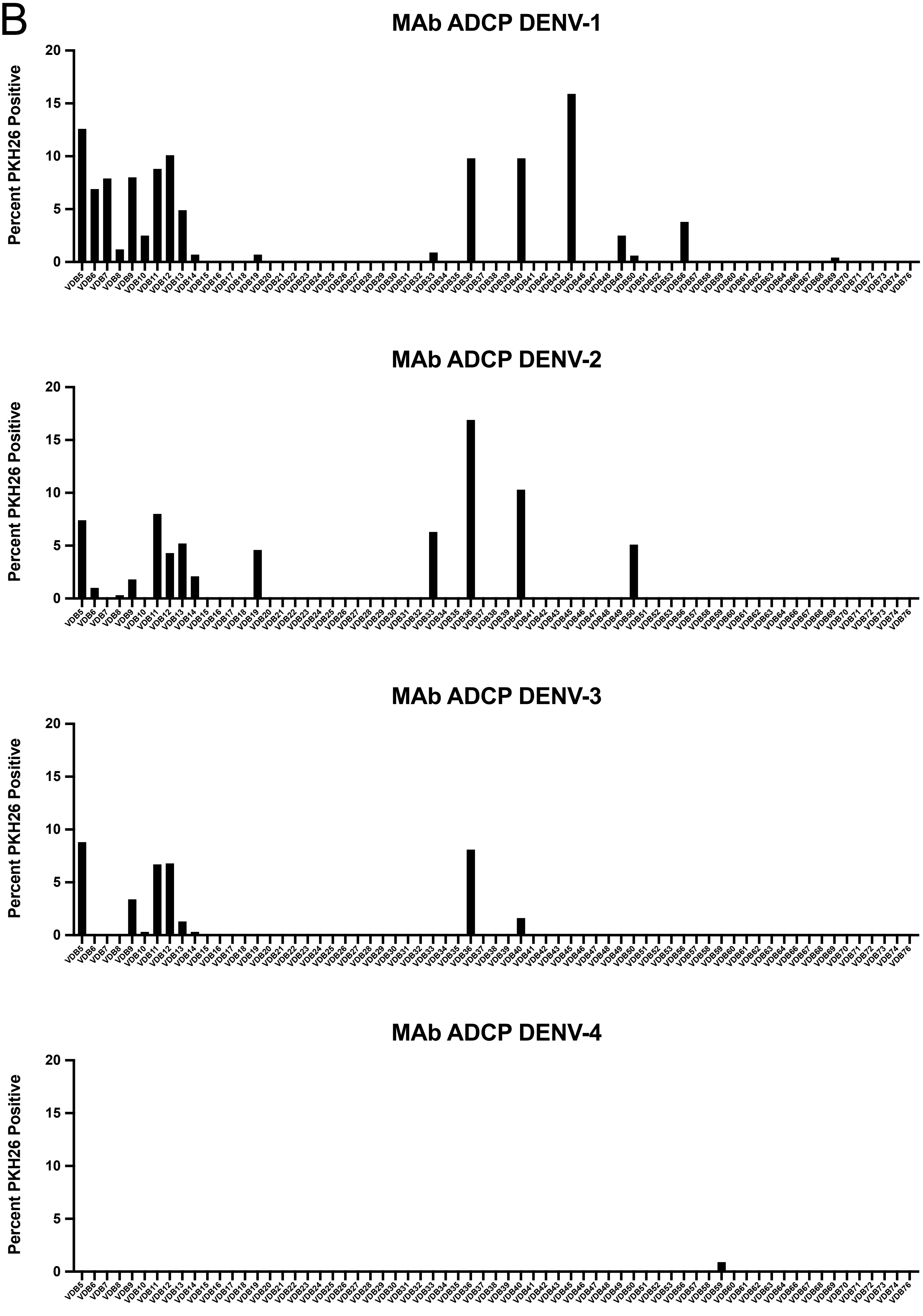

MAb ADCP DENV-1
MAb ADCP DENV-2
MAb ADCP DENV-3
MAb ADCP DENV-4
Percent PKH26 Positive
0
5
10
15
20
