# Supplementary material for: Applications and insights from continuous dengue virus infection in a stable cell line
Source: Front Immunol. 2025 Jun 24;16:1618650. doi: 10.3389/fimmu.2025.1618650 (PMC12234473; doi:10.3389/fimmu.2025.1618650)

Supplementary Figure 10: **A.** Gating strategy (below) for the ADCD assay and determination of C3 deposition on the surface of DENV-infected cells. The example below shows initial gating for identification of DENV-infected cells (2H2+) and then subsequent staining for C3 deposition of the cell surface using an anti-C3-FITC MAb after incubation with SC2 plasma. **B.** Shows gating on 2H2- cells (uninfected cells) matching the format of Figure 10. Note the low level of C3 deposition on the cells compared to that seen on infected cells in Figure 10.

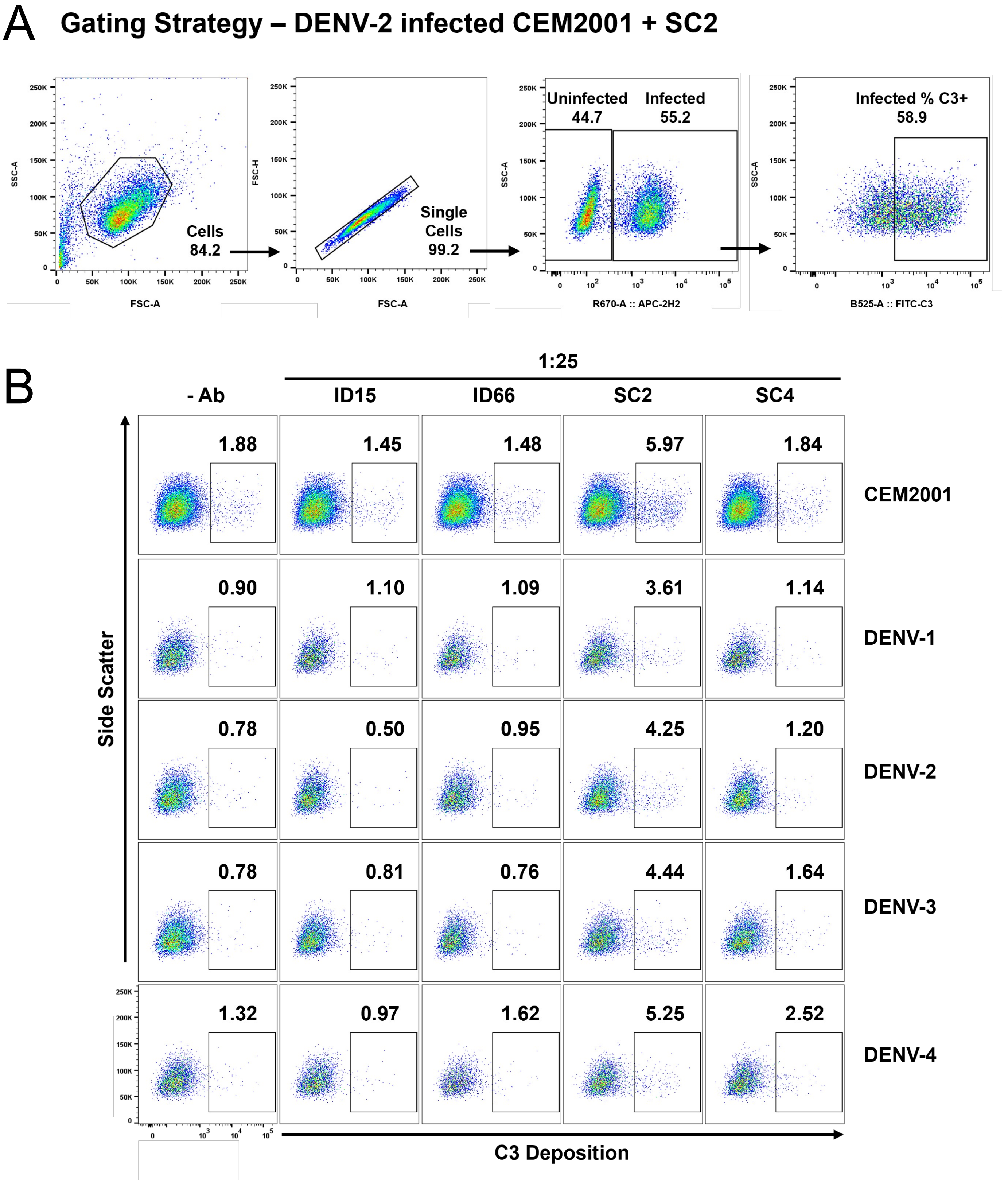

Supplement: Supplementary file 10 [file DataSheet10.pdf]
